# The Cradle-to-Cradle Life Cycle Assessment of Polyethylene terephthalate: Environmental Perspective

**DOI:** 10.3390/molecules27051599

**Published:** 2022-02-28

**Authors:** Muhammad Tamoor, Nadia A. Samak, Maohua Yang, Jianmin Xing

**Affiliations:** 1 CAS Key Laboratory of Green Process and Engineering, State Key Laboratory of Biochemical Engineering, Institute of Process Engineering, Chinese Academy of Sciences, Beijing 100190, China; tamoor@ipe.ac.cn; 2 College of Chemical Engineering, University of Chinese Academy of Sciences, 19 A Yuquan Road, Beijing 100049, China; 3 Chemistry and Chemical Engineering Guangdong Laboratory, Shantou 515031, China

**Keywords:** cradle-to-cradle life cycle, mainland China, PET water bottles, recycling scenarios, environmental impact, sustainability

## Abstract

Over the last several years, the number of concepts and technologies enabling the production of environmentally friendly products (including materials, consumables, and services) has expanded. One of these ways is cradle-to-cradle (C2C) certified^TM^. Life cycle assessment (LCA) technique is used to highlight the advantages of C2C and recycling as a method for reducing plastic pollution and fossil depletion by indicating the research limitations and gaps from an environmental perspective. Also, it estimates the resources requirements and focuses on sound products and processes. The C2C life cycle measurements for petroleum-based poly (ethylene terephthalate) (PET) bottles, with an emphasis on different end-of-life options for recycling, were taken for mainland China, in brief. It is considered that the product is manufactured through the extraction of crude oil into ethylene glycol and terephthalic acid. The CML analysis method was used in the LCIA for the selected midpoint impact categories. LCA of the product has shown a drastic aftermath in terms of environmental impacts and energy use. But the estimation of these consequences is always dependent on the system and boundary conditions that were evaluated throughout the study. The impacts that burden the environment are with the extraction of raw material, resin, and final product production. Minor influences occurred due to the waste recycling process. This suggests that waste degradation is the key process to reduce the environmental impacts of the production systems. Lowering a product’s environmental impact can be accomplished in a number of ways, including reducing the amount of materials used or choosing materials with a minimal environmental impact during manufacture processes.

## 1. Introduction

Human actions have generated a variety of environmental issues during the previous few centuries. More than a dozen environmental factors have had an enormous impact on human life, such as the following: climate change, glacier melts, the depletion of the ozone layer, deforestation and the loss of biodiversity, soil degradation, and water pollution. Our lives depend on the quality and conditions of the environment, and we have a moral obligation and a sense of responsibility to our grandchildren to address these challenges. Because we have made so many synthetic and chemically complex chemicals, there are no natural analogues. They are harmful since we do not know how long they take to degrade or what effect they have on living things. One such substance is plastic. Sadly, due of its usefulness for humans, it is widely employed all across the world. Approximately 3.8 × 10^2^ Mt (million metric tons) of plastic were produced globally [1,2], needing approximately 6% of global crude oil production [3], and generating annual waste equal to the annual production, due to the streams from previous years [4]. In addition, the global plastics market is predicted to double during the next two decades [5]. PET is one of those plastics that we cannot live without. There are several uses for it in packaging, textiles, and films, as well as vehicles, electronics, and more. Water bottles and soda bottles are common examples of translucent plastic containers. A person can learn more about PET and discover why it is a perfect choice for so many different applications. Also, its blends are formed with different thermoplastics and thermosets, processing requirements, and of course these benefits make PET an important polymer worldwide. The research studies related to PET are a necessity of the current time in order to better handle it within society and the environment. PET is composed of different elements, which are shown in Table S1.

PET is obtained by a reaction between a di-acid and a di-alcohol, followed by several stages of polycondensation. The bis-hydroxyethyl-terephthalate (BHET) monomer can be produced either by trans-esterification of dimethyl terephthalate (DMT) or ethylene glycol (EG) or by the direct esterification of terephthalic acid (TPA) and EG [6]. Since 1970, the path by direct esterification of TPA and EG has progressively been adopted [7]. Only the PET process will be further considered in this study.

Furthermore, because over 99% of plastics are derived from fossil fuels, their rapid expansion will place additional strain on the earth’s already limited non-renewable resources. This new backdrop of an ecologically-concerned society has aided in the development of innovative plastics solutions with lesser environmental implications [8]. ‘Plastic’ is now acknowledged as a universal term that encompasses the vast diversity of polymers currently available on the market, each with a unique chemical composition, mechanical properties, manufacturing methods, and raw material feedstock (e.g., petroleum vs. renewable resources) [9]. This is what has allowed them to be used in practically every human activity. Plastics include chemo-physical, mechanical, optical, and biological properties that, due to their adaptability to a wide range of tasks, have contributed considerably to their rising use, making them more ubiquitous in daily life [10]. For this type of policy-context decision-making, environmental LCA is a typical and widely applied tool [11].

Environmental concerns are causing consumers and producers to pay more attention to their purchases. This motivates the production and promotion of more environmentally sustainable products (raw to final product). In order to cope with the problem of manufacturers and consumers in determining the environmental performance of materials/products, C2C philosophy can be utilized. The philosophy of C2C is a novel paradigm used to model products and services that are advantageous in terms of economic, well-being, and environmental sustainability in order to establish a tenable world [12]. It presents a hopeful vision of the future, in which items are fundamentally changed in order to benefit both human beings and their surroundings. This idea is based on increasing the positive benefits and reducing the negative consequences (as in LCA) [13]. A transformation to a circular economy necessitates a shift in consumer and business paradigms toward utilization rather than ownership. The elimination of single-use bottles in the short term, especially in applications with strict hygiene requirements, is a significant challenge. As a result, there is still a need to look for alternatives to single-use bottles that are more sustainable. Although nothing positive can be accomplished by making little tweaks to a fundamentally flawed system, the negative aspects can be mitigated slightly [14]. The purpose of C2C for the built environment is to encourage smart designs that have a positive synergistic interaction with the natural world. Drawing inspiration from natural flow systems where the sun is the major source of energy and process waste is digested to produce nutrients for other biological processes is the only way to do this [12]. Biological nutrients and technical nutrients are highlighted, with the former comprising the nature-sphere and the latter the techno-sphere, which are both digested and regenerated by nature. The goods should be developed as such [15]. Several tools and approaches have recently been developed to help corporations to analyze their products’ environmental performance [16], including carbon [17] and water footprints [18], LCA [19], or more recently, the product environmental footprint (PEF) approach of the European Commission [20]. Many of these procedures are documented by international standards [21].

The cascade model and supra-recycling, in which a material or product that is no longer useful or valuable is turned into a material or product of equal or more usefulness/worth, are emphasized in this ideology [22]. This framework raises three points of view. Residue from one process may be used as a source of nutrients for another, arguing that the goal should be to develop systems with products that other processes can use as nutrients, rather than minimizing waste [15]. The toxicity of the item is also taken into consideration in this approach [23]. Sustainable energy refers to the utilization of renewable sources of energy, such as solar power, in order to meet the energy needs of industrial activities [12], rather than the use of fossil fuels, which devastates the locations where these materials have been kept, to extract the abiotic resources. One of the three concepts, referred to as “celebrating variation”, is based on the assumption that diversity enhances a product or system’s resilience and robustness in a rapidly changing environment [24]. As a result, variety is required to boost a system’s resistance. Concentrating on a single criterion may result in instability and imbalance in the larger context [25]. The application of these principles of C2C can be described in such a context.

If the multilayer structure of the product prevents it from being recycled, the idea of incinerating it and recovering the energy is suggested. It is important to keep in mind that the container may be disassembled and its components separated into pre-established cycles as part of the C2C’s eco-design (a vital pillar).

A study will be conducted to investigate how the values change when wind, water, or solar energy substitutes electricity. Green energy credits are also rated. The sensors’ active time could be modified to save electricity. A saving mode on the specified microcontrollers minimizes the intelligent system’s electricity usage. Conductive polymer sensors’ low operating temperatures are also recommended.

Assuming all of the providers of the raw materials are Chinese, they contribute to local job creation, as does the production plant in Spain, which creates training and employment opportunities.

The management of plastic materials’ end-of-life is a critical issue in the market viability of plastic materials (for example, PVC has been taken off the market of plastic bottles for lack of an effective recycling system). Biopolymers, such as non-renewable plastics (from oil and natural gas), confront technical hurdles in terms of end-of-life management, despite the fact that their end-of-life channels are more numerous, particularly when very large volumes are at stake, such as in the goods market. This is why it is critical to address this issue.

Life cycle analysis is an effective analysis tool to assess the environmental impacts of a product or system at all stages of its life. It also assesses the environmental effects of a product over its full lifecycle. An environmental impact assessment (EIA) is a comprehensive examination of a product’s whole life cycle, beginning with the extraction of the raw materials and ending with the product’s final disposal. The pre-consumer phases are responsible for the bulk of environmental consequences, including global warming, acidification, and air pollution. This research uses a complete track LCA analysis and aims to see the environmental profile of petrochemical polyethylene terephthalate (PET) water bottles. We will also discuss the environment-friendly process of recycling and degradation as end-of-life to again become a useful resource. The LCA can be performed by examining a product’s environmental footprint from raw materials to production (cradle-to-gate) or by analyzing the entire product life cycle, including product disposal (cradle-to-grave).

Firstly, C2C is a philosophy that sets out defined goals at the start of the process, whereas LCA advises identifying “nodes” and measuring the environmental impact of a product. By definition, products that consume resources and produce waste at the end-of-life are contaminated by the C2C approach [13]. The cradle-to-cradle brand is owned by McDonough Braungart Design Chemistry (MBDC) in Charlottesville, Virginia, and used under license by the Environmental Protection Encouragement Agency (EPEA) in Hamburg, Germany [26]. The C2C Product Innovation Institute in California certifies the goods. Unlike the LCA, which is owned by no one, and was produced by a worldwide community of experts. The UNEP-SETAC lifecycle provides some of the coordination, while ISO 14040/44 provides the applicable framework. In terms of communication and marketing, C2C involves the client by purchasing certified things, whereas LCA communicates the redesign hotspots. The LCA compares functionally comparable items, whereas the C2C compares a product at various phases of optimization. According to C2C, the optimal source is determined by local conditions, whereas LCA determines the best source based on the environmental effect calculations [13].

The crucial question is, *why is the China region considered for PET LCA?* It is due to the fact that “the China waste management market is extremely competitive and is predicted to develop at a CAGR of more than 7% during the forecast period of 2019–2024. In 2016, the People’s Republic of China was the largest market for plastic garbage, accounting for over 60% of global imports. In 2016, the G7 countries accounted for over half of all plastic garbage exports to China. In addition, China is the world’s second-largest producer of waste. Until January 2018, when the Chinese government banned the import of 24 categories of solid waste, including mixed paper and plastics, it was a net importer of a massive amount of rubbish from other nations. In addition, the waste industry consumed approximately 2.1 × 10^2^ tons in 2017, representing a 4.7% increase over 2013. China imports a large amount of recycled materials as industrial raw materials. As a result, market participants in this business have a lot of room to grow”.

This work aims to analyze cradle-to-cradle life cycle analysis of PET plastic. In order to examine the environmental sustainability and impacts of PET packaging (as an example “drinking water bottles”), through the use of the “LCA”. Different studies regarding LCA of packaging are present in the literature but with limited aspects and conditions. We have, for the first time, introduced the LCA model with the necessary seven processes that occur in the life cycle of PET bottles. In the case of a circular economy in this model, the LCA loop is connected by introducing the stage of grave-to-cradle. This model is based on the China region by considering the need for LCA study for such a large market in the world. According to our research and literature study, this model has never been considered before. Bottles of the same mass were included in our investigation. Environmental impacts, in terms of distance and energy usage, are also considered. This article reports the uses of an efficient track LCA model.

## 2. Materials and Methods

A specific analytical protocol is used to analyze the product/process/entire service’s life cycle or lifetime, from the time its raw materials are obtained to their end-of-life, and management in the future, beginning with extraction, production, process development, consumption, and disposal of the product/process/service [27]. To put it another way, LCA assists the industrial sector in restructuring its technologies/processes in order to lessen the environmental impacts [28]. Unlike the environmental impact assessment, LCA focuses on broadening traditional assessment precincts and providing a greater scope to environmental assessment as a whole [29]. System inputs and outputs (e.g., natural resources, waste, and by-products) are collected and analyzed in order to create quantitative data on their probable environmental impacts [30]. LCA holds that all phases of a product’s or activity’s life cycle are liable for its environmental impacts. According to several studies, packaging material production is a hotspot in the product’s life cycle, as well as EIAs [31]. According to the European Union, LCA is the greatest tool for assessing a product’s possible environmental impact [32]. OpenLCA is open-source and free software for sustainability and LCA. It competes with commercial LCA tools, such as SimaPro, GaBi and Umberto, but has important distinctions. Figure S1 depicts the International Standards Organization (ISO) framework for LCA (ISO 1997; ISO 1998; ISO 2000; ISO 2006) with four distinct methodological processes, namely (a) goal and scope definition, (b) inventory analysis, (c) impact assessment, and (d) interpretation [33,34,35]. Each has a very important role in the assessment. The LCA approach is very adaptable and tailored to the specific needs of the study. LCA results will be customized and implemented in accordance with the LCA methodology. For an authentic LCA, the following stages of integration are necessary, with crucial variables to be considered and clearly described [36,37]. In Section 2, the results of each of the main components of LCA methodology applied to the packaging systems (PET bottles) under study are presented.

The life cycle of PET comprises the upstream and downstream life cycles. The downstream (cradle-to-grave stage) life cycle considers the manufacture of PET from raw materials to the distribution and consumption of PET bottles for different applications. The upstream (grave-to-cradle stage) life cycle includes post-consumption of PET bottles, which includes waste collection, recycling, and treatment of non-recyclable waste.

*Environmental footprint in C2C life cycle* = *Extraction and resin production* + *Preform production* + *Final form production* + *Distribution* + *Waste collection* + *Recycling* + *Treatment of non-recyclable waste*

The major contributors to the impact footprints in these streams are the raw material extraction, resin production, and final product production processes. The primary goals of conducting an LCA in our scenario are to research the manufacturing process, environmental footprints, and reduction in plastic pollution.

### 2.1. Life Cycle Assessment

#### 2.1.1. Goal and Scope and Boundaries

Goal and scope are concerned with conceptualization, comprehension, and clarification of possible LCA metrics. One of the most crucial steps of LCA methodology is the goal and scope stage, which is often overlooked [27]. One of the most important steps in conducting a research project is determining its scope. The system boundary, functional unit, allocation, and cut-off criteria are some of the major factors in this phase of LCA methodology that can have a significant impact on the study’s results. It also includes determining the objective of the investigation, which might be based on the study and comprehension of a product’s life cycle, the creation of manufacturing methods, and the utilization of findings for marketing reasons.

The purpose of this research is to evaluate the life cycle and environmental performance of PET bottles manufactured in mainland China (Goal 1)., to analyze the life cycle for better recycling and degradation of PET waste, and the production of environment-friendly products and processes. The phrase “PET waste” refers only to PET bottles, it does not include the plastic cap, label, or any leftover residues in the bottle (Goal 2). As indicated in Section 2.1.4, the purpose of this LCA study also includes several management strategies for PET waste by analyzing several scenarios (Goal 3).

*Scope:* The chemical industry is the most energy-intensive sector within the business, with polymers being its most important product. Synthetic plastics have gained a lot of attention in the previous decade. It is motivated by the prospect of increased anthropogenic greenhouse gas (GHG) emissions as well as a reduction in raw material supply from fossil fuels. Another reason for this heightened focus is the continued high price of oil. As impact categories, the scope also covers global warming, acidification, ozone depletion, aquatic eutrophication, non-renewable energy, land occupation, respiratory organics, respiratory inorganics, and aquatic ecotoxicity.

For this study, system’s boundaries are defined as those points at which material flows and unit operations are deemed to be part of the study’s scope (ISO Standard 14040). With a certain system boundary in mind, a specific LCA variant can be used. Using different variations of a product at different stages or processes might also widen the scope of LCA’s application. The LCA variations have different goals, but they all try to assess environmental sustainability.

**Figure 1:**
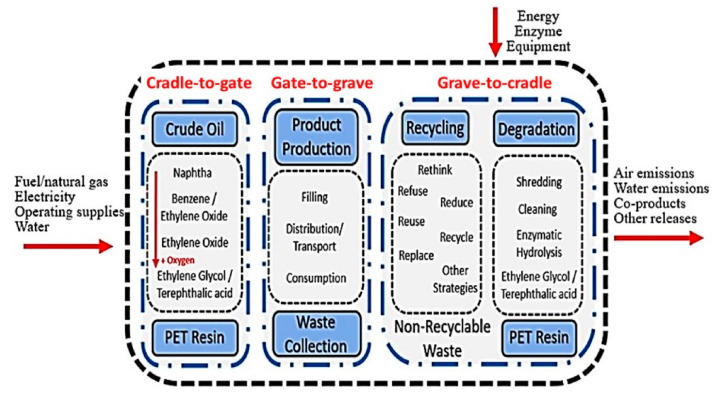
Cradle-to-gate, gate-to-grave, and grave-to-cradle life cycle flow chart of plastic bottles of PET.

*System boundaries:* Beyond “cradle-to-grave” the system encompasses the critical stage when waste is discharged by consumers and ends when its worth and utility are recovered or at its minimal safe disposal. The presented technology is based on the closed-loop recycling of PET bottles. The system’s border encompasses everything from raw materials through PET waste collection and transportation, recycling or disposal, and the replacement of fossil-based PET granulate (by the esterification of EG and TA process). The system’s inputs are as follows: material flows include PET waste, diesel fuel for collection and transportation vehicles, and process equipment; not included materials include the creation of facilities and waste management installations; and energy flows include electricity/fuel for process operations. The system’s outputs are as follows: emissions from collection and transportation, direct emissions from waste management procedures, manufacturing processes, indirect emissions from facility building, electricity/heat production, and PET manufacture using fossil fuels. All certification levels of C2C do not cover the whole life cycle of a product, which might cause an error to be shifted between stages of the life cycle [38]. The complete life cycle of a PET bottle can be divided into various stages. Figure 1 shows the step-by-step life cycle of the C2C model with system boundaries.

This division of the life cycle helps us in the identification of hot spots in the product life cycle, contributing to the environmental burdens generated in each category and the resource depletion.

*Cradle-to-gate:* The raw material required for the production of PET resin and its extraction with blown bottles is considered during this stage. This stage also considers the transportation/shipping of the materials to the gate of the production facility.

*Gate-to-grave:* The filling, distribution, and consumption of bottles take place in this stage. In the last part, the network of the informal collection system collects a major percentage of the product consumed/used by the end-users.

*Grave-to-cradle:* Recycling and degradation take place in this stage. In this stage, used bottles are collected and then segregated, packed, and processed for further application. The various resources, such as electricity, utilities, and chemicals needed for PET resin, bottles production, distribution recycling, and degradation, are mapped as the LCI data.

*Functional Unit:* The functional unit’s aim and scope phase is “the measure that permits performance quantification of the product system and serves as the reference for all of the input/output material/energy flows” [34]. A fair comparison of environmental consequences can only be achieved if the system boundaries and functional unit are properly defined in the research process. No more than assigning the flow of energy and resources (material) into and out of a product process/system is involved in allocation; therefore, the amount of energy and resources (materials) that end up in the corresponding product is a crucial component that ultimately contributes to the uncertainty. The lack of particular data necessitates the use of trustworthy assumptions, which may also have certain limits. The practitioner must establish cut-off criteria for the quantity of energy flow, material flow, or environmental significance associated with the unit processes or product system to exclude the impacts below the cut-off criteria [39]. The functional unit defined is one ton (1 t) of PET polymer.

#### 2.1.2. Inventory Analysis

This phase focuses on obtaining the necessary data to accomplish the study’s objectives (ISO Standard 14040). The life cycle inventory has the following two components: process quantification and the presentation of a unified process flow graphic concerning the functional sources (LCI). The flow of energy and raw materials through the system, as well as the emissions (to air, water, and soil) of the product or process, must be accounted for throughout its full life cycle. When analyzing a complex system, it is possible to break it down into smaller subsystems and processes. Emissions, energy consumption, and material flow are all documented for each procedure. This data may be adjusted to illustrate the entire life cycle of a product or process [40]. This study focuses on the production of petroleum-based plastic bottles. The obtained data is evaluated, transformed, sorted, and presented based on the predicted functional unit and system boundaries.

The hydrocarbon-based polymer’s life cycle begins with the extraction of crude oil and the cracking of the extracted oil. Following crude oil extraction, including resin manufacture, related gases are converted into ethylene, naphtha to benzene and ethylene oxide, ethylene oxide to ethylene glycol, and finally to PET [41].

In terms of data selection, the following hierarchy is applied:

1. Primary data are recommended because they are site-specific, as well as representative of geographic, technological, and historical scopes;
2. In the absence of primary data, either secondary data from the literature or modified LCI data based on site-specific information are employed. As a result, these statistics are only partially particular to the facility under evaluation and are blended with proxy data, such as average data from similar businesses;
3. Data from generic LCI databases are the least desirable alternative. These data are not particular to the facility under evaluation and are chosen based on the best approximation.

PET polymer is manufactured through the use of crude oil, as the primary raw source in the production of plastic, crude oil is employed. One liter of petrol (7.6 × 10^−1^ kg) requires approximately 9.0 × 10^−1^ kg of crude oil, with one kilogram of crude oil equaling 44 MJ/kg. This yields a plastic-to-petroleum ratio in kilograms per liter [42]. Purified terephthalic acid (PTA) and mono-ethylene glycol are produced during the monomer manufacturing stage (MEG). When paraxylene is oxidized with acetic acid and cobalt as a catalyst, PTA is formed. It takes 6.6 × 10^−1^ kg paraxylene, 4.3 × 10^−1^ kg of water, 4.7 × 10^−1^ kWh of energy, and 3.9 MJ of heat to generate 1 kg of PTA (Ecoinvent, 2008). Chemically, MEG may be made by reacting the first-step intermediate derivative of ethylene oxide with water and then turning it into MEG. Ethylene oxide is more expensive to create than MEG, since it needs 8.3 × 10^−1^ kg ethylene, 4.6 × 10^−1^ kg of liquid oxygen, and 3.3 × 10^−1^ kWh of energy to make 1 kg of MEG (Ecoinvent, 2008). Using electricity and heat data from MTEC’s Thai databases, Ecoinvent updated the inventory figures for both monomers in 2009. PET resin is created by combining PTA, MEG, and a catalyst. The major processes in the manufacturing process include raw material preparation, esterification, pre-polycondensation, and polycondensation. In order to make 1 kg of PET resin, 8.7 × 10^−1^ kg of PTA, 3.5 × 10^−1^ kg of MEG (Indorama Venture Public Company Limited, Bangkok, Thailand, 2013), 3.8 × 10^−1^ kWh of energy, and 6.3 MJ of heat (Ecoinvent, 2008) are needed.

Stretch-blow molding is used to turn polymer pellets into plastic bottles. Polymer type, polymer mass (measured by the weight of injected packages), and machine model and capacity determine the amount of power needed to process a given amount of polymer. In Bangkok, Thailand, a genuine production factory provided inventory data for plastic bottle weights and injection electricity needs. Based on 1 kg of PET bottle manufacture, 1.1 kg of PET resin and 2.3 kWh of power are required [43].

If necessary, information regarding the discovered inputs and outputs can be refined in a second cycle. If there is no information on the volume of a release or the actual substance emitted, an educated guess is made based on logical factors. Further and more extensive examinations were carried out in circumstances where such an assumption dominated the LCA result, and some of the values were reassessed. If the rough assumption has no effect on the outcome, it serves no purpose and is retained in the inventory.

OpenLCA scientific tool is used by academics all over the world. It is a highly competitive, full-fledged, and innovative LCA/sustainability modeling software. The OpenLCA scientific team is frequently asked about scientific research that has been undertaken using OpenLCA, thus, they decided to provide a list of peer-reviewed journal papers relating to OpenLCA. Analysis of “cradle-to-cradle” PET LCA has been performed with OpenLCA. Different studies from the literature with the facts and figures of the present model related to the region of mainland China are considered in detail for the important environmental impacts. The focus of the work is to become closer to green technology and a safe environment.

**Figure 2:**
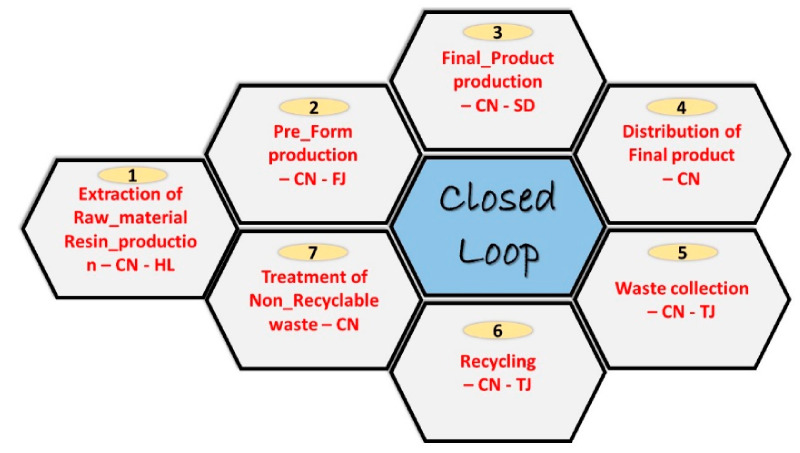
Mainland China PET plastic model graph from cradle-to-cradle in OpenLCA.

The bottle manufacturing includes different processes, as mentioned in above Figure 2. These processes take place in various areas of China. The following information was obtained through a Google search based on the model. Transportation for PET resin involves transit from Heilongjiang to Qingdao, where raw-material to resin production facilities are considered, when modelling the LCI. Then, for the perform production process, the resin is considered to travel from Qingdao to Xiamen. For the production of the final product, it is assumed to be transported back to Qingdao to be blown and for water addition. The final product is distributed across the major areas, such as Qingdao to Harbin, Qingdao to Urumqi, and Qingdao to Lijiang. After the usage, the post-consumer waste product is collected for the recycling site in Tianjin. In last stage, the sorted non-recyclable waste is assumed to be the maximum distance that was traveled between these locations. Table 1 displays all of the distances entered into the model.

**Figure 3:**
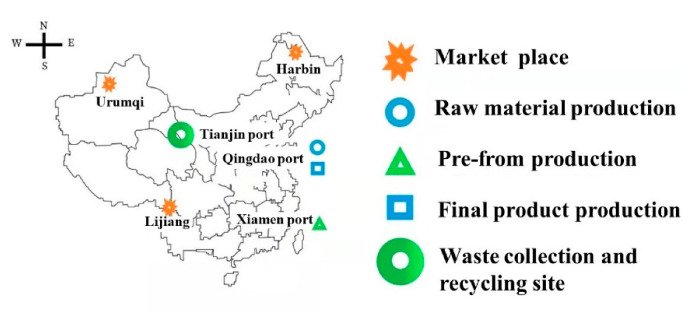
Different locations and included process map.

In the present model, we have attempted to consider all of the key cities of the country, i.e., Qingdao (east), Urumqi Market (west), Harbin Market (north), Lijiang Market (south). The locations on the map are seen in Figure 3.

Songliao Basin is a huge terrestrial sedimentary basin in Northeast China, bound by the Greater Khingan, Lesser Khingan, and Changbai mountains. It stretches about 2.6 × 10^5^ km^2^ and is traversed by the Songhuajiang and Liaohe rivers in the provinces of Heilongjiang, Jilin, and Liaoning. For the extraction of crude oil, the approximate area considered is 4.1 × 10^3^ km^2^ (Google search). For the production of PET resin in the model, the company considered is Qingdao Yatong Chemicals Company limited. It is a high-tech firm based in the seaside city of Qingdao that is involved in the research, manufacturing, and supply of plastic raw materials and chemical raw materials. Inputs and outputs for production machinery and infrastructure are also provided. To the greatest extent possible, the mass and energy flows of these systems are displayed on a per-unit basis. Following inputs, assumed regarding the extraction of raw material and resin, production is mineral extraction land (4.1 × 10^3^ km^2^), energy (kWh), lorry (in terms of time and distance), crude oil (4.4 × 10^4^ MJ), catalyst (2.7 × 10^−1^ kg) [44], mono-ethylene glycol (7.2 × 10^2^ kg), purified terephthalic acid (6.6 × 10^2^ kg) and water (2.5 m^3^) [43]. While the emissions into the environment include are arsenic (7.6 × 10^−5^ kg), biological oxygen demand (BOD5, 14 kg), cadmium (3.4 × 10^−5^ kg), chloride (14 kg), chemical oxygen demand (COD, 45 kg), lead (3.0 × 10^−4^ kg), methane (1.3 kg), nitrate (1.5 × 10^−1^ kg), particulates (>2.5 μm and <10 μm, 1.1 × 10^−1^ kg) [45], phosphate (3.7 kg) [46], sulfur dioxide (1.6 kg), total organic carbon (TOC, 40 kg) [45], industrial waste (3.0 × 10^−2^ ton) [46], and CO_2_ for 1 ton of resin production (5.2 × 10^3^ kg) [47]. Because the amount of antimony used in PET preparation ranges from 1.0 × 10^2^ to 3.0 × 10^2^ mg/Kg, a 1-L container may contain 3–9 mg of antimony. We are considering the max limit of catalyst i.e., 3.0 × 10^2^ mg/Kg. For the production of the preform of bottles, the company Quanzhou Jupin Group Co., Ltd. in Fujian, with exportation from the sub-provincial city location Xiamen, is considered. They have a production capacity of 8.0 × 10^5^ pieces/month [32]. For making the preforms of the bottles, the following inputs are considered: energy (kWh), lorry, and resin (1 ton). The output industrial emissions into air, water, and soil are, ethylene oxide (4.9 × 10^−1^ kg) [48], formaldehyde (1.0 × 10^2^ kg) [48], methane, trichlorofluro-, CFC-11 (3.5 × 10^−5^) [48], sulfur dioxide (73 g) [46], sulfur trioxide (1.1 kg) [48], toluene (42 kg) [48], tri-ethylene glycol (7.8 × 10^3^ kg) [48], preform production (0.97 t), and for 1 ton, produce 12.2 kg CO_2_ [46]. For the final product production, i.e., bottles, the company Qingdao Greesing Supply Chain Co., Ltd. in Shandong, with exportation from the coastal city location Qingdao, is considered. They have a production capacity of 1.0 × 10^8^ pieces/month [12]. For the production of bottles, the input and outputs are energy (kWh), lorry, preform (1 ton), and drinking water (42 m^3^) [49]. Due to transportation, the CO_2_ emission for 1 ton produce 12.2 kg CO_2_. While, for the industrial waste into the soil and/or water, the emission of phosphate and sulfur dioxide are 1.7 × 10^−1^ kg and 3.3 kg, respectively [46]. After the bottles production, they are filled with water and transported to the three different locations inside China, i.e., Harbin, Urumqi, and Lijiang. These distribution locations are selected in such a way that they influence all directions in China. The input conditions regarding the filled water bottles distribution around the China include energy (kWh), lorry, empty bottles, and water weights, which are 1 ton and 4.5 × 10^2^ tons, respectively. Due to transportation, emissions for 1 ton produce 12.2 kg CO_2_. While, after consumer usage, the produced waste of the bottles is accounted to be 0.9 ton.

For the waste collection and recycling process, the company Hesoo Technology Tianjin Co., Ltd. in Tianjin is considered [12]. Since the 1980s, Chinese companies have imported foreign solid waste, particularly plastic, paper, and metal garbage, that might be used as raw materials in manufacturing and construction [50,51]. According to UN figures, more than 70% of global plastic waste, and 37% of global waste paper, including that from the United States and Europe, were sent to China in 2015 [52]. The primary goal of such waste imports is to compensate for a lack of indigenous resources as a result of a fast expanding economy [53]. In the previous two decades, there has been an increase in waste imports. Input assumptions for the waste collection are land occupied (5.9 × 10^2^ km^2^), energy (kWh), lorry, and waste (1 t) [54]. From the total area of Tianjin (1.2 × 10^4^ km^2^), we have considered only 5% for the waste collection. Due to transportation, emission of CO_2_ for 1 ton produced 12.2 kg.

PET bottle collection systems differ by nation due to differences in local conditions, which include the technical design of the recycling system, government legislation, and recycling culture. The collection process for recycling in China involves informal dealers for PET bottles, similar to Brazil [55]. During the study period, the total amount of PET bottle recycling in China was 78 million tons (Mt). Among these, 29 Mt of discarded PET bottles (37% of total recycling) were imported from outside, accounting for 40% of total global exports. We will consider only the Chinese own waste recycle i.e., 78 Mt − 29 Mt = 49 Mt waste PET that is recycled [56], this means during 2000–2018 in China, 2.72 Mt recycled PET waste of the Chinese population was generated. Input assumptions for the recycling are energy (kWh) and sorted waste (1 t). Emissions due to the transportation are 25% of CO_2_ emitted from 1 ton polymer production, which will produce 1.3 × 10^3^ kg of CO_2_. It means recycled PET (rPET) contributes 25% CO_2_ into the environment, compared to virgin PET production [47]. Then, 3.0 × 10^−1^ tons polymer (rPET) produces 3.9 × 10^2^ kg CO_2_. China produced 63 million tons of plastic in 2019, with a recycling rate of around 30% [57].

Chinese plastic recycling plants directory offers a listing of Chinese companies that recycle plastic waste into new commodities. China is home to 76 plastic recycling factories [58]. Table 2 shows the details regarding the recycling plant that is selected in the present LCA model.

Today, the increased national and global focus on resource efficiency and emission reduction is forcing the globe to enhance its waste management system. One important goal is to implement the waste hierarchy outlined in the European Waste Framework Directive 2008/98/EC [59,60]. The waste hierarchy consists of five levels, mentioned in Figure S2. First, waste generation should be eliminated to the greatest extent practicable. Second, whenever technically possible, unavoidable waste should be repurposed. Third, garbage that cannot be reused directly should be recycled. Fourth, non-recyclable waste should be recovered, such as through energy recovery. Last, but not least, only leftover waste fractions, for which none of the above-mentioned treatments are available, are legally permitted to be landfilled.

The Chinese MSW management tax is a flat-rate billing scheme; the MSW disposal fee per home in Chongqing’s main districts is RMB 3/month (USD 0.4/month) [61]. Domestic waste disposal fees in Beijing range between RMB 2 an RMB 3 per family each month, depending on residency status. The monthly rubbish collection fee in Guangzhou is RMB 10 per home. Furthermore, households in different provinces are exempt from paying waste disposal taxes. The waste is used for power generation in China, at a different place, with pyrolysis technology, as shown in Table S2.

For the non-recyclable waste treatment, the method of biodegradation is considered. With the help of chemo-enzymatic hydrolysis, the non-recyclable (mechanically) waste needs treatment. For this purpose, its location is not specifically mentioned because the calculations are performed based on the experimental work on the laboratory-scale. Input assumptions considered here regarding the degradation are land area (5.1 × 10^3^ m^2^), energy (kWh), enzyme (6.5 × 10^4^ kg), lorry (in terms of time and distance), non-recyclable waste (7.0 × 10^−1^ t), additives (Ca^2+^, Mg^2+^), and surfactants. Half additives are assumed in comparison to surfactants [62]. With consideration of the 97% recovery rate of monomers, we obtained total recovered monomers of 6.8 t [63]. In Table 3 the mean net weight used during the processing is shown.

#### 2.1.3. Impact Assessment

The life cycle impact assessment (LCIA) stage is concerned with comprehending and evaluating data in order to determine its relevance in terms of potential environmental repercussions in a system. Emissions and resources are categorized into numerous impact categories, then converted to common impact units, and made comparable throughout this phase. The term “life cycle impact assessment” is commonly used to describe this phase (LCIA) [40,64]. LCIA is normally carried out following ISO 14044: 2006, which specifies four processes, two of which are required and two of which are optional. The obligatory phases are (i) selection of impact categories and classifications and (ii) characterization. The optional steps are (iii) normalization and (iv) weighing. As an integral part of the LCA process, selecting impact categories and classifying them are key tasks that must be completed to properly allocate elementary flows based on an inventory of compounds and their impact on a wide range of environmental issues. The impact of each emission/element on the environment is calculated and quantified as part of the LCIA process in terms of the equivalents of a reference substance. Raw material phosphate equivalents can be expressed in kg PO_4_, based on the eutrophication impact category [65].

Various methodologies for assessing life cycle impact are available in the literature [66]. Using OpenLCA, the CML analysis method was used in the LCIA. Following many modifications and enhancements, the Leiden University Center for Environmental Science produced an “Operational guide to the ISO standards” in 2001 that describes exactly how LCA should be undertaken for a process/product/project, in accordance with the ISO standard regulations. An extensive list of chemicals (natural resources and emissions to nature) was included in the handbook to help with the impact assessment process. Various climate, environmental, health, and resource depletion factors are among the CML methodology’s midway categories. Various modeling aspects are discussed and adopted in a way that is mutually trustworthy and relates to the management of time, space, non-linearity’s, economic, social, and technical systems [67]. The method discusses the data uncertainties, but they are not quantified. Each of the countries in the EU, and the rest of world, has its normalization factors for CML. It is explained, but not numerically modeled, how midpoints and endpoints are linked together.

The categories analyzed included global warming (GWP100), photochemical oxidation, abiotic depletion, ozone layer depletion (kg CFC-11 eq.), eutrophication (kg P eq.), acidification (kg SO_2_ eq.), human toxicity (kg 1, 4-DB eq.), freshwater aquatic ecotoxicity, terrestrial ecotoxicity (kg 1, 4-DB eq.), and marine aquatic ecotoxicity. The impact of global warming is due to CO_2_, CH_4_, and N_2_O, being converted to CO_2_ equivalency to create a total of global warming potential. Photochemical oxidation was accounted for using toluene and formaldehyde. Abiotic depletion is caused due to the depletion of fossil fuels and minerals. The emission of chlorofluorocarbons (CFCs) and halons causes ozone layer depletion. SO_2_ equivalents, which comprised NO_x_ and NH_3_, were used to account for acidification. Eutrophication was caused by the flow of nutrients, primarily nitrogen and phosphorus, into bodies of water. Human toxicity is due to the presence of heavy metals, such as arsenic. The terrestrial ecotoxicity is dominated by emissions of heavy metals and sulfuric acid. In freshwater aquatic ecotoxicity, the most influential factors that affect it are formaldehyde and cadmium. The marine aquatic ecotoxicity is dominated by formaldehyde, arsenic, and cadmium. Human toxicity, as a result of resource usage, is the category with the largest impact. Because they are made from nonrenewable raw resources, this has an impact.

#### 2.1.4. Interpretation

In LCA, the interpretation part involves the evaluation of either inventory analysis or impact assessment results with a defined purpose and scope to arrive at recommendations and conclusions (ISO Standard 14040). This phase includes the analysis and alignment of outcomes with the goals and scope phase. There is a thorough review of the entire cycle, and any issues are addressed sequentially [68]. It specifies the following components of the interpretation phase (ISO Standard 14040: 2006):

*Identification:* The interpretation results are designed to identify the most essential themes, based on the study’s goal and scope;

*Evaluation:* The study’s comprehensiveness, sensitivity, and consistency are evaluated. To establish and improve the dependability of the results, the results should be presented in a clear and easily-understood manner;

*Results, restrictions, and advice:* The results of the study will be presented in the form of conclusions, recommendations, and limitations.

When using a quantitative tool to evaluate an individual’s environmental footprint, the assumption might be challenged. The degree to which a C2C product’s designated attributes may be quantified by an LCA depends on the measurement’s percentage. In other words, how detailed can the attributes be stated, rather than merely conceptually [69]. However, if a future scenario has been decided upon, an LCA can be used to evaluate future technology. In other words, the C2C of a product can be evaluated using data that predicts the likely future C2C design solution. This data’s measurability is dependent on its ability to foresee future outcomes [70]. The LCA may also be used to calculate a C2C product’s established positive footprint. This would be used to calculate the benefits of the solutions (idea evaluation), not the disadvantages [71].

Units are assigned to each category based on the influence it has on the overall system. However, each stage/bottle may only be compared to another stage/bottle that has the same categories (for example, global warming, pollution, etc.), i.e., GWP100 in resin production and GWP100 in recycling waste [72,73]. The data may be analyzed in terms of each product’s contribution to the overall environmental impact, as well as the sum of each process’s affects, to compare the total effects of each product. This means that data may reveal both the product that has the largest environmental effect and the process that takes place inside each process to make that product. It was analyzed and compared for each impact category [74]. Table 4 shows the total consequences of water bottles, while Section 3 shows the effects of the different production steps.

Natural resource exploitation and anthropogenic sources (such as fossil fuels, automobiles, and smokestacks) regularly leak greenhouse gases (GHGs) into the atmosphere, where they eventually become part of the Earth’s atmosphere and help gather heat to warm the globe [75]. The PET bottle contained 1.6 × 10^−1^ kg CO_2_ eq in this category, with an impact factor of 4.6 × 10^3^ kg CO_2_ eq/kg. Terrestrial ecotoxicity refers to the discharge of hazardous compounds (such as formaldehyde) into the environment that are toxic to organisms [76]. The PET bottle had an impact factor of 9.4 × 10^−1^ kg 1, 4-DB eq/kg, and an impact result value of 1.1 × 10^2^ kg 1, 4-DB eq. Photochemical air pollution is caused by the oxidation of hydrocarbons and nitrogen oxides by light. Its side effects include eye discomfort, vegetation damage, reduced sight, rubber cracking, and color fading [77]. The photochemical oxidation impact factor was 1.2 kg C_2_H_4_ eq/kg for the PET bottle, with an impact result value of 9.2 × 10^1^ kg C_2_H_4_ eq. There are no prominent changes in the abiotic impact category that occurred during the C2C life cycle assessment of 1 ton PET bottles. Human toxicity occurs due to the existence of ethylene oxide and formaldehyde [78]. This causes skin problems when in contact and, due to inhalation, causes dizziness or suffocation. It had an impact factor of 1.4 × 10^4^ kg 1, 4-DB eq/kg for the PET bottle, with an impact result value of 8.1 × 10^3^ kg 1, 4-DB eq. Terrestrial acidification is characterized by changes in soil chemical properties following the deposition of nutrients (namely nitrogen and sulfur) in acidifying forms. It is a global threat to plant diversity and is mainly caused by the atmospheric deposition of acidifying compounds [79]. It had an impact factor of 5 × 10^−1^ kg SO_2_ eq/kg for the PET bottle, with an impact result value of −6.1 kg SO_2_ eq. The ozone layer, which is found in the Earth’s atmosphere, protects living organisms from the sun’s powerful UV radiation. Cancer and cataracts can be caused by exposure to ultraviolet light, which can degrade organic compounds. The ozone layer, which is made up of three oxygen atoms bound together, can be damaged by certain chemicals. Ozone-depleting substances are commonly referred to as such since they contain anthropogenic compounds, such as methane, trichlorofluoro-, and CFC-11 [80]. The PET bottle was discovered in this investigation to have an impact factor of 1 kg CFC-11 eq/kg in this category, with an impact result of 3.6 × 10^−5^ kg CFC-11 eq. Almost always, eutrophication in freshwater habitats is caused by excessive phosphorus and nitrogen. It alters the ecosystem structure by increasing algae and aquatic plant growth, decreasing fish populations, and generally degrading water quality [81]. It had an impact factor of 1.2 kg PO_4_ eq/kg for the PET bottle, with an impact result value of 4.0 kg PO_4_ eq. Freshwater aquatic ecotoxicity is due to the emission of poisonous substances released into the environment during the product production process. These emissions have the potential to harm aquatic ecosystems and degrade ecosystem quality. Aquatic ecotoxicity for the PET bottle has an impact factor of 8.3 kg 1, 4-DB eq/kg and an impact result of 9.7 × 10^2^ kg 1, 4-DB eq. While marine aquatic ecotoxicity was considered due to the presence of formaldehyde, arsenic, and cadmium, with impact factor of 1.3 × 10^6^ kg 1, 4-DB eq/kg and an impact result of 2.5 × 10^2^ kg 1, 4-DB eq. These categories were mainly due to the extraction of raw material, resin, preform, and bottle final manufacturing processes.

In the end-of-life options, the mechanical recycling process and chemo-enzymatic hydrolysis are assumed. According to world-meter elaboration of the most recent United Nations data, China has a total area of 9.6 × 10^6^ km^2^ and a current population of 1.4 × 10^9^ as of 2021. Since the mid-1990s, China’s recycling collection system has changed dramatically. Until the mid-1990s, the government collected all recyclables as part of the planned economy. Recyclers are those who collect and recycle PET bottles. Small community waste-buying facilities, medium/large redemption centers, and recycling businesses are all examples [82]. The government discontinued offering waste redemption as a result of the reform and opening-up program of the 1980s. Thus, informal recycling collection was delayed until the mid-1990s as a result. This trend is still evident today. In the absence of government oversight, scavengers’ health, competition among informal sector participants, and environmental issues have arisen [83]. As a response, Beijing’s central and municipal governments have made an effort to standardize and streamline recycling.

The Ministry of Commerce published a pilot program in 2006 to collect recyclables, which was announced by the central government. Recycling Management was published in 2007. In 2006, Beijing initiated a pilot program and established a work plan for a formal recycling system. Small community waste-buying depots are popping up all over China [84]. Improved PET bottle recycling, such as bottle-to-bottle technology, is also needed. Scavengers, itinerant garbage purchasers, small community waste-buying depots, medium/large redemption depots, and recycling corporations are the five types of recyclers in Beijing. The first three are collectors of post-consumer PET bottles, whereas the last two are RPET (recycled PET) resin dealers who act as a bridge between PET bottle collectors and end-users. The five types of recyclers have the following recycling practices [85]. Scavengers collect recyclables (including PET bottles) from garbage cans in order to make a profit and survive. PET bottles were openly collected by scavengers on streets, shopping malls, subway stations, residential districts, and dumps or transfer stations. Itinerant waste collectors collect PET bottles from residences and sell them to medium or large redemption depots. PET bottles are collected for formal recycling by small community waste-buying depots. Small community waste-buying depot owners have a 5–10 m^2^ space in which to gather recyclables from the neighborhood. If customers call, they will also do door-to-door recycling. Currently, only a few enterprises are qualified (i.e., have pollution control equipment under local government oversight) to use rPET resins. The small manufacturers employ rPET resins and do not have pollution control equipment. The price of PET bottles sold to small rPET resin users in Beijing is market-based.

Only two recycling firms washed, de-labeled, and removed caps from PET bottles. This thorough recycling of PET bottles enhances the quality of rPET resins, which may then be used to manufacture high-efficiency items. In Beijing, there are only a few of these recycling businesses. The utilization efficiency of rPET resins in China is significantly lower than in the US and Japan, and there is plenty of room for improvement. Figure S3 depicts the material flow for Beijing’s PET bottle recycling collection system.

Due to current environmental control loopholes and increasing demand for recovery materials, the informal sectors of China have begun low-cost waste recycling activities. According to [86], informal sectors are associated with major environmental and health difficulties, as well as a lack of formal recyclers and remanufactured product safety issues. Prohibiting or competing with the informal sector is ineffective, therefore, they must work together in the future. 

## 3. Results and Discussion

However, because this article did not conduct a consequential LCA, and it was not the aim of this project to estimate uncertainties in the LCI data, the technological gap was not taken into account. The following important points related to the OpenLCA tool during the evaluation of the results are considered:

1. No allocation method is considered because only one product (bottles) LCA is studied;
2. No regionalized LCA is present in the literature for the current study related to the region of China;
3. Life cycle impact assessment method considered here is CML.

**Figure 4:**
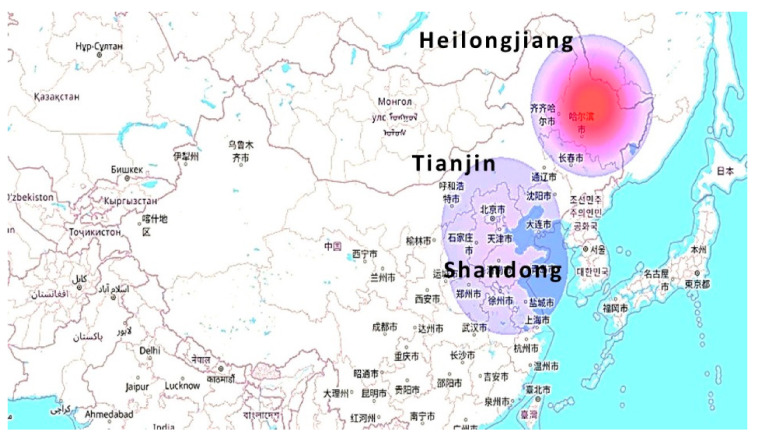
Carbon dioxide emission effects.

The LCA of PET bottles from C2C has also been assessed through the Keyhole Markup Language (KML) maps. These maps show the impact of the important essential flows at different locations for the environmental effects. Figure 4 represents the CO_2_ emission into the environment. The effect is more prominent due to the mineral extraction process in the Heilongjiang province, amounting to 6.0 × 10^3^ kg. While for Shandong and Tianjin province it is minor when compared with them, i.e., 5.2 × 10^2^ kg and 4.0 × 10^2^ kg respectively.

**Figure 5:**
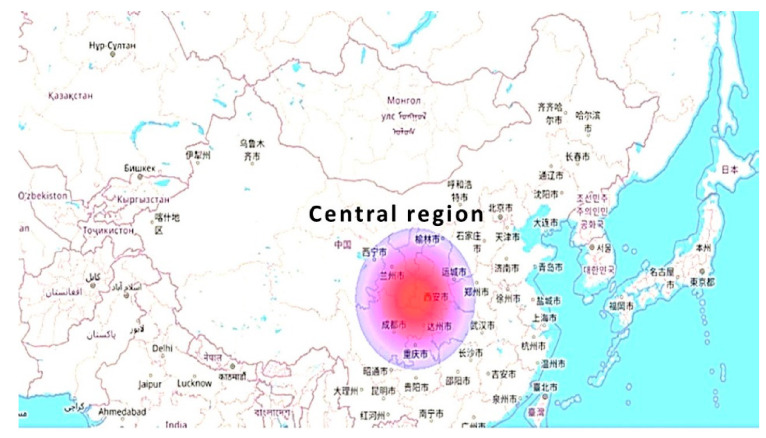
Energy consumption effects.

In Figure 5, the maximum energy consumption effect is clear. It can be seen that the need for energy consumption is maximum (2.2 × 10^10^ MJ) in the central region, in comparison to the extremes.

**Figure 6:**
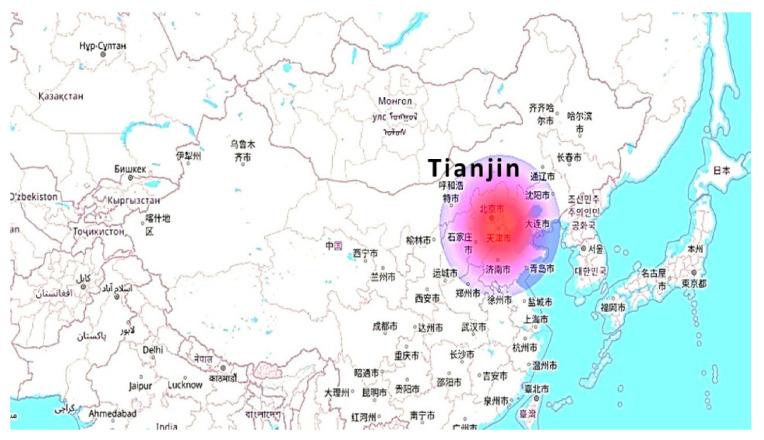
Land occupation effects.

Figure 6 depicts the land occupation for the production processes of the plastic production from C2C. Due to the waste collection and recycling processes, maximum land is needed in the Tianjin region, i.e., 5.9 × 10^8^ m^2^.

**Figure 7:**
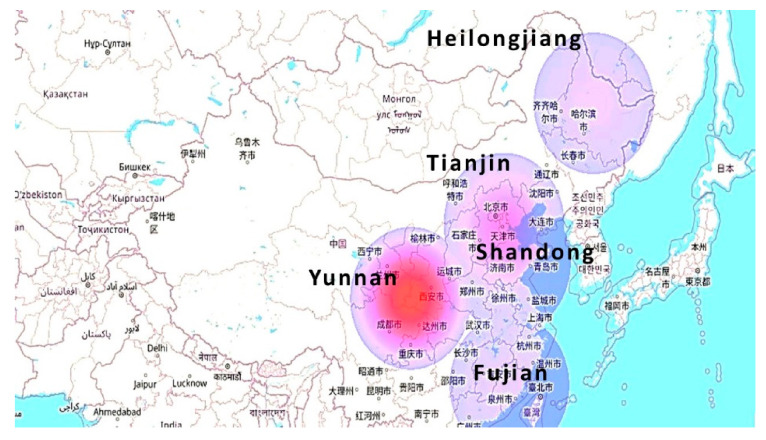
Lorry effects.

The transportation effect due to lorries is revealed in Figure 7. In the map the maximum effect (1.5 × 10^4^ t km) is due to the waste collection and recycling process, because of the transportation towards the Tianjin region from other regions. The effect is calculated in terms of both the weight and the distance traveled. For the other regions, Fujian (1.9 × 10^3^ t km), Shandong (1.9 × 10^3^ t km), Heilongjiang (2.3 × 10^3^ t km), and Tianjin (6.7 × 10^3^ t km).

**Figure 8:**
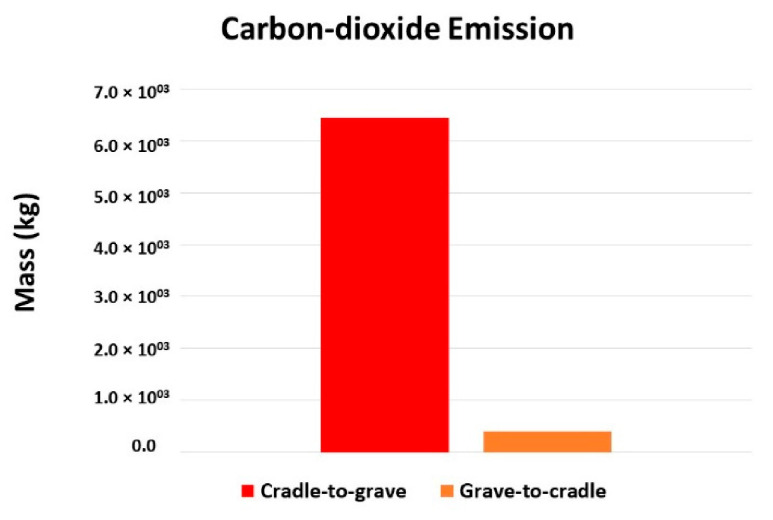
Comparison of carbon-dioxide emission into the environment.

The model calculations for group data are also discussed in brief. The term “cradle-to-grave (C2G)” represents data from the extraction of raw materials to the consumption by consumers. While the term “grave-to-cradle (G2C)” represents data from the waste collection to the production of recycled raw material. According to Figure 8, the CO_2_ emissions from C2G and G2C differ 6.0 × 10^3^ kg at maximum. This is happening because of the mineral extraction, bottle production, and the transportation of the goods before and after consumption.

**Figure 9:**
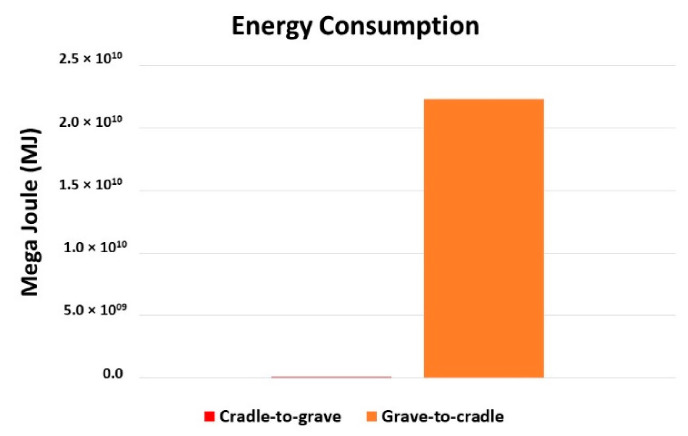
Comparison of energy consumption of product during LCA.

Figure 9 presents the comparison of energy consumption between stages C2G and G2C. The electricity, or heat energy, is maximum for the G2C, i.e., 2.2 × 10^10^ MJ. In the G2C stage, waste product goes through mechanical recycling, cleaning, shredding, melting, and again converting into resin formation, therefore, it needs a lot of energy consumption when compared to C2G. In the C2G stage, the processes include the extraction of raw material to resin and then the production of bottles. In real life, it is a common saying “making a new setup requires less time and money, while demolishing and rebuilding needs more time, money, and efforts”. This is the main drawback of the C2C approach. However, by utilization of renewable energy, and through research in the biodegradation of plastics, we can find more possible ways to reduce the cost, time, and efforts. 

**Figure 10:**
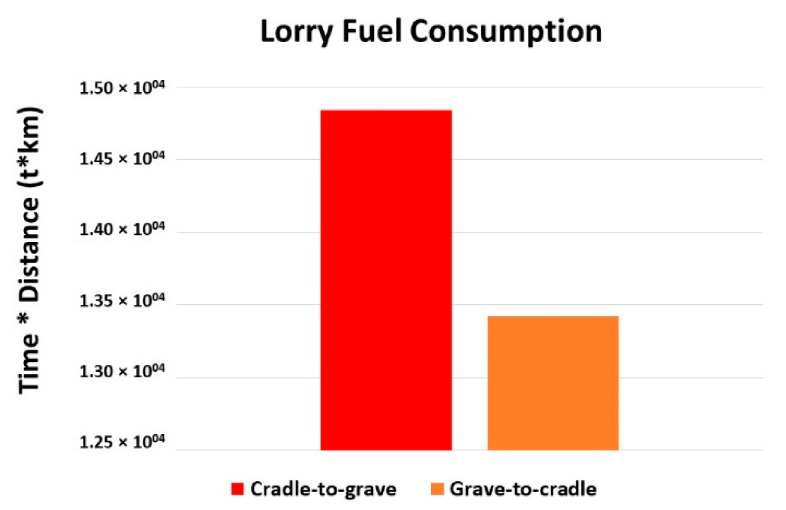
Comparison of lorry fuel consumption during LCA of product.

Figure 10 examines the lorry fuel consumption from C2G and G2C. The fuel consumption due to transportation differs less, i.e., 1.4 × 10^3^ t km.

The results are calculated in order to access the environmental pollution and impacts due to the production of plastic bottles until 2050. In 2019, according to “Plastic the Facts—2020” [87], the demand for the PET plastic-type is about four million tons i.e., 16% distribution of Europe in the world’s plastic production, while it states that China has a 31% contribution globally. This implies that, in 2019, China was contributing PET of approximately 7.8 million tons. In 2050, according to “The future of PET packaging to 2025”, Smithers report [88], an annual growth rate of 3.7% is pushing the consumption of PET, with the help of the mathematical formulation of a geometric sequence (p_n_) and geometric sum (P_n_), as follows:

p_n_ = a r^(n−1)^, r > 1 (1)

where, n = 32, a = 7.75 and r = 1.0372. This implies, from Equation (1),

p_32_ = 24.02 (2)

P_n_ = a (r^n−1^)/(r − 1),

from Equation (2),

P_32_ = 670.44

**Figure 11:**
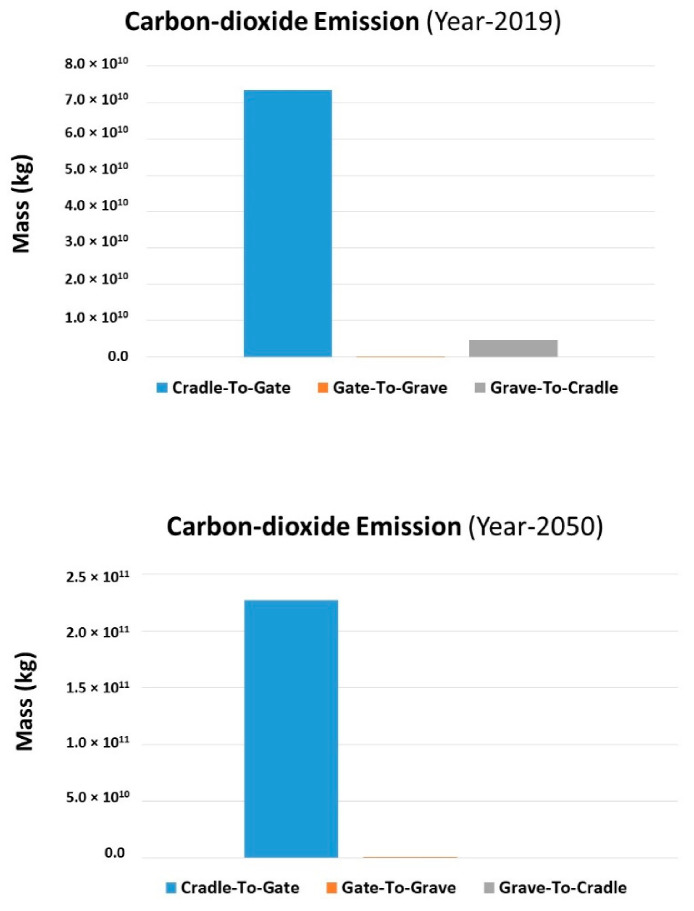
Comparison of Carbon-dioxide emission in 2019 and 2050.

The accumulative value for the PET consumption/production from 2019 to 2050 will be 6.7 × 10^2^ million tons and in the year 2050 growth will be 24.02 million tons. The group data comparisons between 2019 productions with 2050 are mentioned below. The effect of greenhouse gas emission comparison for three decades is mentioned in Figure 11. The emission during Cr2Gt and Gr2Cr has a prominent impact for both cases (the year 2019, the year 2050). On the other hand, the impact of Cr2Gt influences more in magnitude. During the Gt2Gr stage, the emission is due to transportation. While the increase in the amount of greenhouse gas from 2019 to 2050 reaches 1.5 × 10^11^ kg. Other consequences of human-caused emissions are contributing to climate change, including extreme weather, food shortages, and a rise in wildfires.

**Figure 12:**
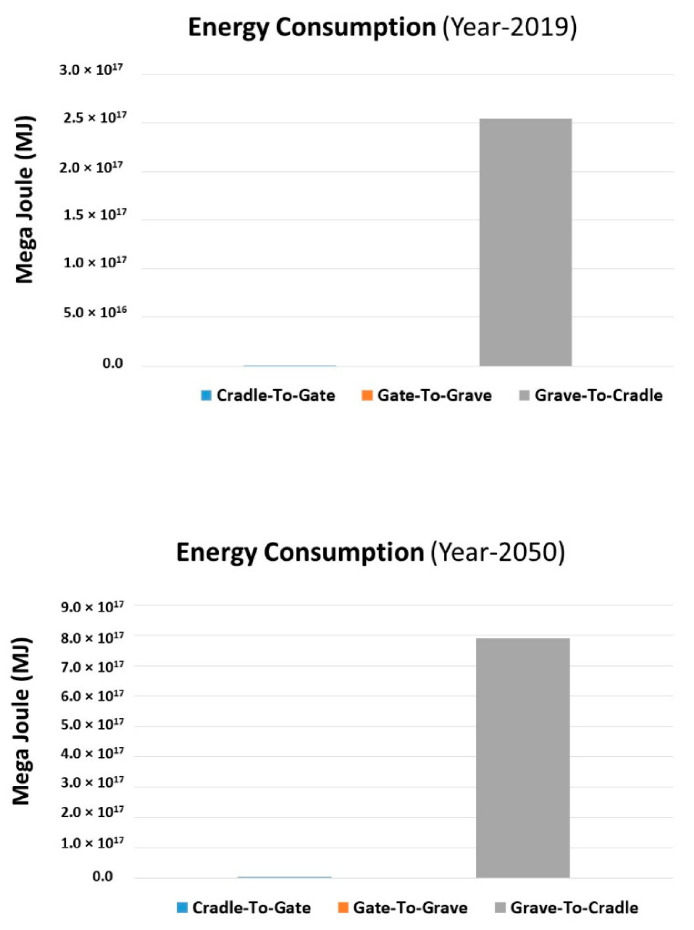
Comparison of energy consumption in 2019 and 2050 of the product during LCA.

The energy consumption during the C2C assessment is depicted in Figure 12. Air pollution, climate change, water pollution, thermal pollution, and solid waste disposal are all issues that are exacerbated by the production and consumption of energy. Fossil fuel combustion releases large amounts of air pollutants into the atmosphere, which is a key contributor to pollution in cities. The energy consumption during Gr2Cr and Cr2Gt has maximum influence for both years 2019 and 2050, while the magnitude of energy consumption impacts more for the Gr2Cr stage. In this stage, the collection, sorting, cleaning, shredding, and the rehabilitation into basic monomers through hydrolysis need an extra amount of energy from the Cr2Gt stage. And the difference in the amount of consumption during Gr2Cr from 2019 to 2050 is 5.4 × 10^17^ MJ. This amount more energy will be required in 2050 for the waste collection and recycling process. 

**Figure 13:**
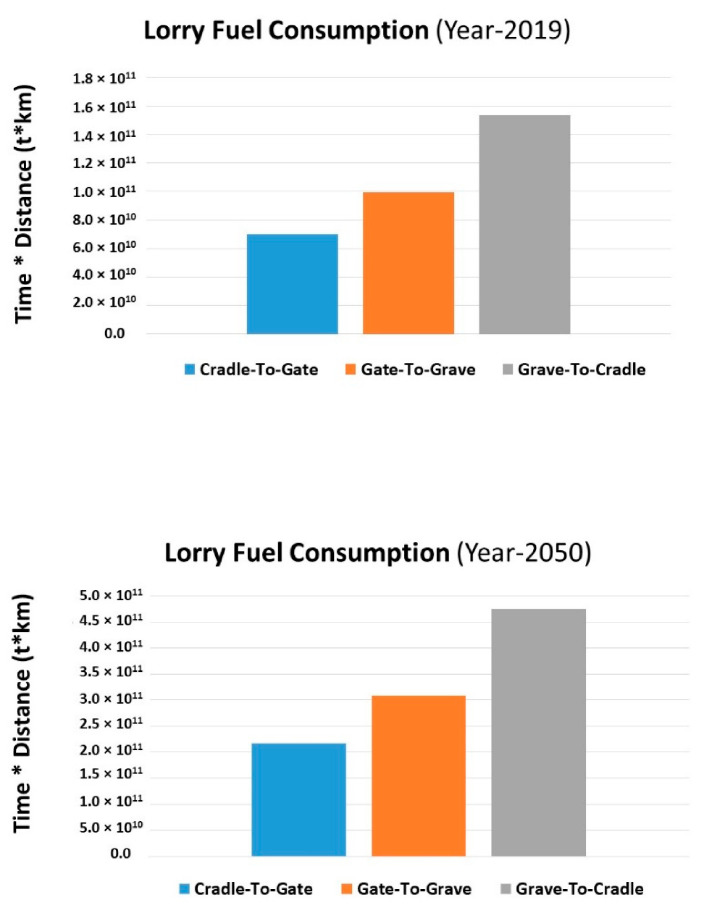
Comparison of lorry fuel consumption in 2019 and 2050 during LCA of product.

Figure 13 depicts a comparison of vehicle fuel use over the C2C life cycle of bottle production over three decades. Fuel vapors and combustion products (carbon monoxide, nitrogen oxides, particulate matter, and unburned hydrocarbons) contribute to air pollution because they release vapors as the gasoline evaporates. Gasoline combustion emits greenhouse gases, such as carbon dioxide. Aside from that, diesel engine emissions damage crops, trees, and other vegetation by raising levels of ground-level ozone. Additionally, acid rain is produced, which enters the food chain via lakes, streams, and rivers. It can also be found in crops, meat, and fish. The lorry fuel emissions during Gr2Cr and Gt2Gr have maximum influence for both years 2019 and 2050, but the Gr2Cr stage has the maximum consumption and emission effects. Basically, at this stage, the maximum transportation process works, and the difference in the amount of fuel used during Gr2Cr from 2019 to 2050 is 3.1 × 10^11^ t km.

## 4. Recommendations for Reducing Environmental Impacts

Sustainability does not require the use of renewable resources. Sustainability depends on how a material is created, used, and recycled, not on its constituent parts. Despite this, bio-plastics have the potential to transform various plastic-intensive sectors into circular economies. Bio-based alternatives are available for almost every fossil-based use, but they are rare, expensive, and do not have many environmental benefits [89]. This study includes a list of recommendations for possible improvements to environmental issues during the “C2C” life cycle. They can assist in obtaining ISO certifications. The following are some suggestions:

- Development and implementation of an enterprise-wide environmental management system;
- Company strategy and goals add the sustainable issues;
- Focus on the production of biodegradable items;
- Improvement in the technologies that are environmentally friendly;
- Reduction in the transportation of the goods through proper management;
- Spreading awareness regarding plastic pollution reduction and a clean/healthy environment;
- Development of the organizations that work on the product LCA and audit the environment;
- Plastic density reduction is the minimum possible for the different applications;
- Special laboratories were established for the technical feasibility, particularly the permeability and mechanical behavior of the film as its thickness is reduced;
- Usage of recycled plastic resins;
- Renewable energy usage, i.e., electricity and heat, from natural sources;
- Optimization of the industrial process and supply chain;
- Usage of mono-layer products for the extension of end-of-life possibilities.

## 5. Conclusions

Plastic waste is one of the world’s most important environmental problems, as it may contaminate land, marine life, and groundwater over time. The environmental challenges involved in managing and handling products and considerations over the entire life cycle of the product need to be taken into account. It is noted that the emission and footprint for the production and transportation of products are significantly larger than the emissions relate to product use and recycling. Although pre-treatment and recycling have been demonstrated to mitigate their impact in C2C assessments, the growing volume of plastic waste and the low percentage of recyclable plastic used underscore the critical nature of the post-treatment of plastic waste. Substituting the C2C strategy for the cradle-to-grave technique can significantly increase the resource recovery.

LCA is a developed technique for determining the magnitude and consequences of environmental contamination, as well as analyzing the avoided loads based on product manufacturing procedures. The LCI provides for extensive analysis of the loads and pollutants allocated at each phase of the processes, as well as the selection of the most appropriate sustainable method. In order to obtain more detailed information about the behavior of the systems under consideration, to optimize process conditions, or to reduce system uncertainty, LCA studies are usually augmented with sensitivity analysis studies. LCA is not limited to environmental assessment; it is considered to be a holistic tool that considers the life cycle cost of the proposed solution and the entire process. Due to a lack of market valuations for emissions and fuel products, dubious findings are generated that are subject to system boundaries, assumptions, and considerations of functional units. This study introduces C2C as a well-studied and reliable technology for reducing the environmental impact of plastic waste post-treatment and underlines the importance of developing a systematic LCA analysis scheme for C2C management. C2C is an advanced waste treatment methodology, and this research could play a vital part in the construction of a strategic plan that takes into account all of C2C’s benefits. The most significant drawback of C2C is its constrained assessment scope, which does not include the product’s whole life cycle or all of the relevant environmental consequences. The recommendations for strengthening the dependability of C2C are based on already known technologies and their actual application.

The entire life cycle in the awarding criteria, the absolute as well as relative (per product unit) amount of used energy, the material and/or a product recycling percentage, no stakeholder consultation, and the involvement and uncertainties are considered. The environmental impact of a product can be decreased in a variety of ways, including by lowering the number of materials used or by selecting materials with minimal environmental impact. An exhaustive list of references is used in this study, which fills the gaps that have been identified, as follows:

- Production and consumption patterns of plastics;
- Research on PET plastic, including end-of-life options;
- More research on the negative impacts of reclaiming plastic waste from the environment, resource savings, harmful effects of manufacturing, and waste processing is also required;
- Recycling education, awareness and technologies need improvement;
- Individual-global-based countries’ research related to LCA studies on plastic waste energy is lacking.

## Figures and Tables

**Table 1:** Distance traveled during each process.

Incineration Type	No. of Plants	No. of Incinerators	No. of Turbine Generators	Total Incineration Capacity (t/d)	Total Power Generation Capacity (MW)
Stoke grate	25	69	46	2.0 × 10^2^	3.6 × 10^2^
Fluidized bed	24	50	39	1.6 × 10^4^	4.2 × 10^2^
Rotary kiln + Pyrolysis	14	32	5	3.5 × 10^3^	2.5 × 10^1^
Total	63	151	90	4.0 × 10^4^	8.0 × 10^2^

**Table 2:** Present model plastic recycling plant based in China [12].

Company Name	Area	Materials Accepted	Recycled Products	Materials Processed (tons/Year)	Capacity (tons/Year)
Hesoo Technolgy Tianjin Co., Ltd.	Tianjin	PET, PP, PS, HDPE, LDPE, ABS	Granules/Pellets	-	5.0 × 10^5^

**Table 3:** Mean weight for each process.

Total Requirements (kg’s)		
Processes	Flows	Amount
Extraction of Raw material and Resin production	PET Resin production	1.1 × 10^3^
Preform production	Preform production	1.1 × 10^3^
Final Product production	Final product	1.1 × 10^3^
Distribution of Final product	Waste	1.0 × 10^3^
Waste collection	Sorted waste	1.0 × 10^3^
Recycling	Non-Recyclable waste	7.0 × 10^2^
Treatment of Non-Recyclable waste	PET Resin Recovery	6.8 × 10^2^

**Table 4:** The impact assessment by standardized categories.

	Impact Assessment: CML 2 Baseline 2000		
Impact Category	Unit	Inventory Result	Impact Result
Global warming (GWP100)	kg CO_2_ eq	3.6 × 10^−5^ kg	1.6 × 10^−1^
Terrestrial ecotoxicity	kg 1,4-DB eq	1.2 × 10^2^ kg	1.1 × 10^2^
Photochemical oxidation	kg C_2_H_4_ eq	1.7 × 10^2^ kg	9.1 × 10^1^
Abiotic depletion	kg Sb eq	-	0.0 × 10^0^
Human toxicity	kg 1,4-DB eq	1.2 × 10^2^ kg	8.1 × 10^3^
Acidification	kg SO_2_ eq	−1.2 × 10^1^ kg	−6.1 × 10^0^
Ozone layer depletion (ODP)	kg CFC-11 eq	3.6 × 10^−5^ kg	3.6 × 10^−5^
Eutrophication	kg PO_4_ eq	4.4 × 10^1^ kg	4.0 × 10^0^
Freshwater aquatic ecotoxicity	kg 1,4-DB eq	1.2 × 10^2^ kg	9.7 × 10^2^
Marine aquatic ecotoxicity	kg 1,4-DB eq	1.2 × 10^2^ kg	2.6 × 10^2^

## Data Availability

All data generated or analyzed during this study are included in this published article (and its supplementary information file).

## Supplementary Materials

The following are available online, Figure S1. Life cycle assessment main components and relation; Figure S2. Waste hierarchy with useful practices; Figure S3. Material flow for Beijing, Chinaߣs PET bottle recycling collection system; Table S1. The elementary composition of the PET plastic [90]; Table S2. Capacity of power generation from MSWI facilities in China [91,92].

## Sample Availability

Samples of the compounds are not available from the authors.
