# Supplementary material for: The Cradle-to-Cradle Life Cycle Assessment of Polyethylene terephthalate: Environmental Perspective
Source: Molecules. 2022 Feb 28;27(5):1599. doi: 10.3390/molecules27051599 (PMC8911646; doi:10.3390/molecules27051599)
Supplement: Supplementary file 1 [file molecules-27-01599-s001.zip › molecules-1608745-supplementary.pdf]

molecules

Supplementary material

The Cradle-to-Cradle Life Cycle Assessment of Polyethylene terephthalate: Environmental Perspective

Muhammad Tamoor [1,2], Nadia A. Samak [1*], Maohua Yang [1,*] and Jianmin Xing [1,2,3,*]

[1] CAS Key Laboratory of Green Process and Engineering, State Key Laboratory of Biochemical Engineering, Institute of Process Engineering, Chinese Academy of Sciences, Beijing 100190, China; tamoor@ipe.ac.cn
[2] College of Chemical Engineering, University of Chinese Academy of Sciences, 19 A Yuquan Road, Beijing 100049, China
[3] Chemistry and Chemical Engineering Guangdong Laboratory, Shantou 515031, China
* Correspondence: nadia@ipe.ac.cn (N.A.S.); mhyang@ipe.ac.cn (M.Y.); jmxing@ipe.ac.cn (J.X.); Tel.: +86-10-6255-0913 (J.X)

Citation: Tamoor, M.; Samak, N.A.; Yang, M.; Xing, J. The Cradle-to-Cradle Life Cycle Assessment of Polyethylene terephthalate: Environmental Perspective. *Molecules* **2022**, *27*, 1599. https://doi.org/10.3390/molecules27051599

Academic Editor(s): Waheed Afzal; Muhammad Usman Azam; Xiangyang Liu

Received: 07 February 2022
Accepted: 24 February 2022
Published: 28 February 2022

Publisher's Note: MDPI stays neutral with regard to jurisdictional claims in published maps and institutional affiliations.

Copyright: © 2022 by the authors. Licensee MDPI, Basel, Switzerland. This article is an open access article distributed under the terms and conditions of the Creative Commons Attribution (CC BY) license (https://creativecommons.org/licenses/by/4.0/).

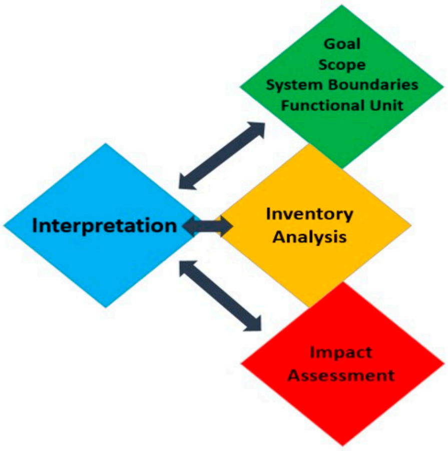

Figure S1. Life cycle assessment main components and relation.

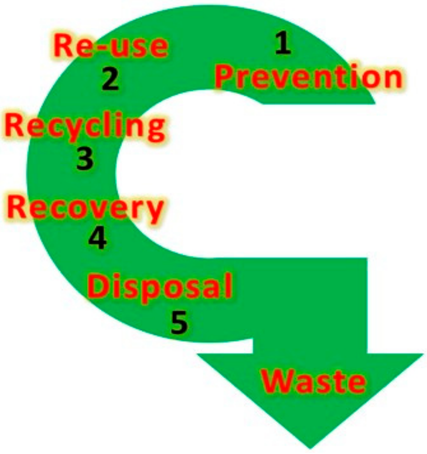

Figure S2. Waste hierarchy with useful practices.

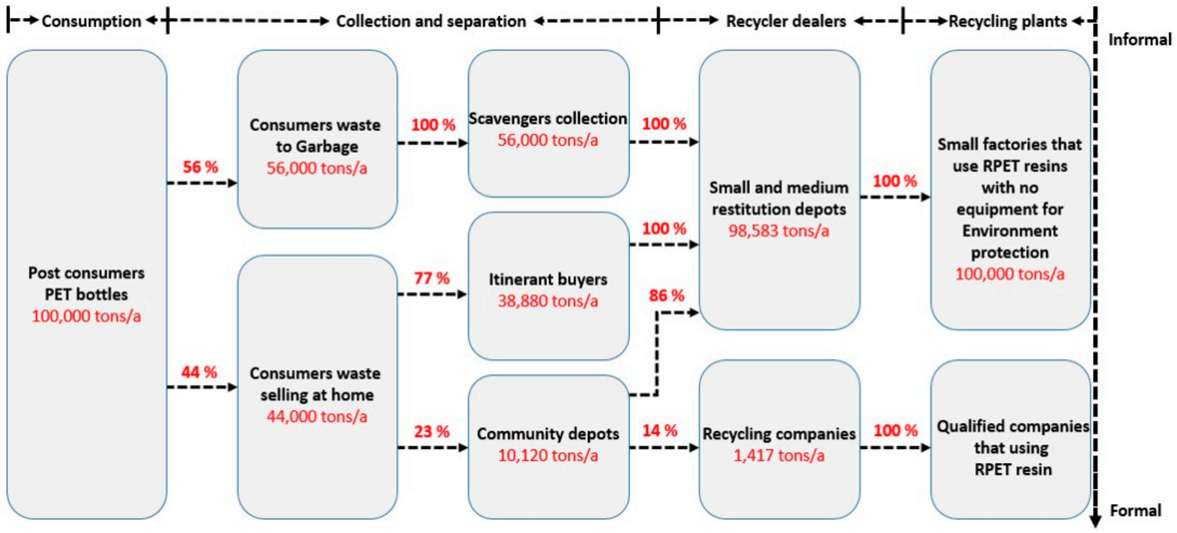

Figure S3. Material flow for Beijing, China's PET bottle recycling collection system.

Table S1. The elementary composition of the PET plastic [90].

**Basic elements**	**Percentage** (%)
Moisture	1.1×10^{-1}
Ash	8.0×10^{-2}
Carbon	6.0×10^{0}
Hydrogen	4.2×10^{0}
Nitrogen	0.0×10^{0}
Sulphur	6.0×10^{-1}

Table S2. Capacity of power generation from MSWI facilities in China [91,92].

**Incineration type**	**No. of plants**	**No. of incinerators**	**No. of turbine generators**	**Total incineration capacity** (t/d)	**Total power generation capacity** (MW)
Stoke grate	25	69	46	2.0×10^{2}	3.6×10^{2}
Fluidized bed	24	50	39	1.6×10^{4}	4.2×10^{2}
Rotary kiln + Pyrolysis	14	32	5	3.5×10^{3}	2.5×10^{1}
Total	63	151	90	4.0×10^{4}	8.0×10^{2}

References

1. Geyer, R.; Jambeck, J.R.; Law, K.L. Production, use, and fate of all plastics ever made. *Sci. Adv.* **2017**, *3*, e1700782.
2. Tiseo, L. *Annual Production of Plastics Worldwide from 1950 to 2020*; Statista: Hamburg, Germany, 2022.
3. Spierling, S.; Röttger, C.; Venkatachalam, V.; Mudersbach, M.; Herrmann, C.; Endres, H.-J. Bio-based Plastics—A Building Block for the Circular Economy? *Procedia CIRP* **2018**, *69*, 573–578. https://doi.org/10.1016/j.procir.2017.11.017.
4. Jambeck, J.R.; Geyer, R.; Wilcox, C.; Siegler, T.R.; Perryman, M.; Andrady, A.; Narayan, R.; Law, K.L. Plastic waste inputs from land into the ocean. *Science* **2015**, *347*, 768–771.
5. Matthews, C.; Moran, F.; Jaiswal, A.K. A review on European Union's strategy for plastics in a circular economy and its impact on food safety. *J. Clean. Prod.* **2021**, *283*, 125263. https://doi.org/10.1016/j.jclepro.2020.125263.

6. Karayannidis, G.P.; Achilias, D.S. Chemical Recycling of Poly(ethylene terephthalate). *Macromol. Mater. Eng.* **2007**, *292*, 128–146. https://doi.org/10.1002/mame.200600341.
7. Dias, D.S.; Crespi, M.S.; Ribeiro, C.A.; Kobelnik, M. Evaluation of the thermal decomposition of blends prepared with poly (3-hydroxybutyrate)(PHB) and recyclable ethylene poly-terephthalate (RPET). *J. Therm. Anal. Calorim.* **2021**, *143*, 3447–3457.
8. Stripple, H.; Westman, R.; Holm, D. Development and environmental improvements of plastics for hydrophilic catheters in medical care: An environmental evaluation. *J. Clean. Prod.* **2008**, *16*, 1764–1776.
9. Walker, S.; Rothman, R. Life cycle assessment of bio-based and fossil-based plastic: A review. *J. Clean. Prod.* **2020**, *261*, 121158.
10. Thompson, R.C.; Moore, C.J.; vom Saal, F.S.; Swan, S.H. Plastics, the environment and human health: Current consensus and future trends. *Philos. Trans. R. Soc. B Biol. Sci.* **2009**, *364*, 2153–2166. https://doi.org/10.1098/rstb.2009.0053.
11. Finkbeiner, M. Product environmental footprint—breakthrough or breakdown for policy implementation of life cycle assessment? *Int. J. Life Cycle Assess* **2014**, *19*, 266–271. https://doi.org/10.1007/s11367-013-0678-x.
12. Ankrah, N.A.; Manu, E.; Booth, C. Cradle to Cradle Implementation in Business Sites and the Perspectives of Tenant Stakeholders. *Energy Procedia* **2015**, *83*, 31–40. https://doi.org/10.1016/j.egypro.2015.12.193.
13. Bjørn, A.; Hauschild, M.Z. Cradle to Cradle and LCA. In *Life Cycle Assessment*, Springer: Berlin/Heidelberg, Germany, 2018; pp. 605–631.
14. Hesselbach, J.; Herrmann, C. *Glocalized Solutions for Sustainability in Manufacturing: Proceedings of the 18th CIRP International Conference on Life Cycle Engineering, Technische Universität Braunschweig, Braunschweig, Germany, 2–4 May 2011*; Springer Science & Business Media: Berlin/Heidelberg, Germany, 2011.
15. Koch, J.; Plehn, C.; Reinhart, G.; Zäh, M.F. Cycle management for continuous manufacturing planning. In *Enabling Manufacturing Competitiveness and Economic Sustainability*, Springer: Berlin/Heidelberg, Germany, 2014; pp. 9–12.
16. Klöpffer, W. Life cycle sustainability assessment of products. *Int. J. Life Cycle Assess.* **2008**, *13*, 89–95.
17. Finkbeiner, M. *Carbon Footprinting—Opportunities and Threats*; Springer: Berlin/Heidelberg, Germany, 2009; Volume 14, pp. 91–94.
18. Wu, M.; Zhang, Z.; Chiu, Y.-W. Life-cycle water quantity and water quality implications of biofuels. *Curr. Sustain./Renew. Energy Rep.* **2014**, *1*, 3–10.
19. Committee, T. ISO 14040: 2006 International Standard—Environmental Management—Life Cycle Assessment—Principles and Framework, *2006*, https://www.iso.org/standard/37456.html (accessed on 28 October, 2021).
20. European Commission; Joint Research Centre. *Product Environmental Footprint Guide*; Institute for Environment and Sustainability: Ispra, Italy, 2012.
21. Finkbeiner, M. From the 40s to the 70s—The future of LCA in the ISO 14000 family. Springer: Berlin/Heidelberg, Germany, 2013; Volume 18, pp. 1–4.
22. Toxopeus, M.E.; De Koeijer, B.L.A.; Meij, A. Cradle to cradle: Effective vision vs. efficient practice? *Procedia CIRP* **2015**, *29*, 384–389.
23. Kausch, M.F.; Klosterhaus, S. Response to 'Are Cradle to Cradle certified products environmentally preferable? Analysis from an LCA approach'. *J. Clean. Prod.* **2016**, *113*, 715–716.
24. Cabot, M.I.; Luque, A.; de las Heras, A.; Aguayo, F. Aspects of sustainability and design engineering for the production of interconnected smart food packaging. *PLoS ONE* **2019**, *14*, e0216555. https://doi.org/10.1371/journal.pone.0216555.
25. Bakker, C.A.; Wever, R.; Teoh, C.; De Clercq, S. Designing cradle-to-cradle products: A reality check. *Int. J. Sustain. Eng.* **2010**, *3*, 2–8.
26. Michael Braungart, W.M. *Cradle to Cradle: Remaking the Way We Make Things*; North Point Press: Berkeley, CA, USA 2002.
27. Curran, M.A. Life-Cycle Assessment. In *Encyclopedia of Ecology*, 2nd Ed.; Fath, B., Ed.; Elsevier: Oxford, UK, 2016; pp. 359–366. https://doi.org/10.1016/B978-0-12-409548-9.09700-1.
28. Brusseau, M.L. *Sustainable Development and Other Solutions to Pollution and Global Change*; Academic Press: Cambridge, MA, USA, 2019; pp. 585–603. https://doi.org/10.1016/B978-0-12-814719-1.00032-X.
29. Židonienė, S.; Kruopienė, J. Life Cycle Assessment in environmental impact assessments of industrial projects: Towards the improvement. *J. Clean. Prod.* **2015**, *106*, 533–540.
30. Grant, T. Life Cycle Assessment (LCA) and Degradable Polymers. In *Degradable Polymers and Materials: Principles and Practice*, 2nd Ed.; American Chemical Society: Washington D. C., USA 2012; Volume 1114, pp. 45–58.

31. Del Borghi, A.; Strazza, C.; Magrassi, F.; Taramasso, A.C.; Gallo, M. Life Cycle Assessment for eco-design of product–package systems in the food industry—The case of legumes. *Sustain. Prod. Consum.* **2018**, *13*, 24–36. https://doi.org/10.1016/j.spc.2017.11.001.
32. Sala, S.; Amadei, A.M.; Beylot, A.; Ardente, F. The evolution of life cycle assessment in European policies over three decades. *Int. J. Life Cycle Assess.* **2021**, *26*, 2295–2314. https://doi.org/10.1007/s11367-021-01893-2.
33. Pennington, D.W.; Potting, J.; Finnveden, G.; Lindeijer, E.W.; Jolliet, O.; Rydberg, T.; Rebitzer, G. Life Cycle Assessment Part 2: Current Impact Assessment Practice. *Environ. Int.* **2004**, *30*, 721–739. https://doi.org/10.1016/j.envint.2003.12.009.
34. Rebitzer, G.; Ekvall, T.; Frischknecht, R.; Hunkeler, D.; Norris, G.; Rydberg, T.; Schmidt, W.P.; Suh, S.; Weidema, B.P.; Pennington, D.W. Life cycle assessment: Part 1: Framework, goal and scope definition, inventory analysis, and applications. *Environ. Int.* **2004**, *30*, 701–720. https://doi.org/10.1016/j.envint.2003.11.005.
35. Klöpffer, W. The critical review of life cycle assessment studies according to ISO 14040 and 14044. *Int. J. Life Cycle Assess.* **2012**, *17*, 1087–1093.
36. Muthu, S.S. Estimating the overall environmental impact of textile processing: Life cycle assessment (LCA) of textile products. In *Assessing the Environmental Impact of Textiles and the Clothing Supply Chain*; Elsevier, Amsterdam, Netherlands, 2014; pp. 105–131. https://doi.org/10.1533/9781782421122.105.
37. Shaked, S.; Crettaz, P.; Saade-Sbeih, M.; Jolliet, O.; Jolliet, A. *Environmental Life Cycle Assessment*; CRC Press: Boca Raton, FL, USA, 2015. https://doi.org/10.1201/b19138.
38. Vercoulen, R. *Impacts of the Cradle to Cradle Certified Products Program*; Cradle to Cradle Products Innovation Institute: Venlo, The Netherlands, 2014; pp. 1–145.
39. UN. Life Cycle Initiative, 2020.https://www.lifecycleinitiative.org/ (accessed on 15 October, 2021).
40. Hellweg, S.; i Canals, L.M. Emerging approaches, challenges and opportunities in life cycle assessment. *Science* **2014**, *344*, 1109–1113.
41. Singh, A.; Rorrer, N.A.; Nicholson, S.R.; Erickson, E.; DesVeaux, J.S.; Avelino, A.F.T.; Lamers, P.; Bhatt, A.; Zhang, Y.; Avery, G.; et al. Techno-economic, life-cycle, and socioeconomic impact analysis of enzymatic recycling of poly(ethylene terephthalate). *Joule* **2021**, *5*, 2479–2503. https://doi.org/10.1016/j.joule.2021.06.015.
42. Gervet, B. *The Use of Crude Oil in Plastic Making Contributes to Global Warming*; Lulea University of Technology: Lulea, Sweden, 2007; pp. 1–8.
43. Papong, S.; Malakul, P.; Trungkavashirakun, R.; Wenunun, P.; Chom-in, T.; Nithitanakul, M.; Sarobol, E. Comparative assessment of the environmental profile of PLA and PET drinking water bottles from a life cycle perspective. *J. Clean. Prod.* **2014**, *65*, 539–550. https://doi.org/10.1016/j.jclepro.2013.09.030.
44. Europe, P. Antimony and Bottled Water, 2019, https://packagingeurope.com/antimony-and-bottled-water/ (accessed on 20 October, 2021).
45. Singh, A. K.; Agarwal, H.; Sinha, R.; Jha, S. K.; Prakash, A. Life Cycle Assessment of Polyethylene Terepthalate (Pet) Bottles. *ELK Asia Pac. J.* **2016**, 1–7.
46. Gironi, F.; Piemonte, V. Life Cycle assessment of polylactic acid and polyethylene terephthalate bottles for drinking water. *Environ. Prog. Sustain. Energy* **2011**, *30*, 459–468.
47. Maga, D.; Hiebel, M.; Aryan, V. A comparative life cycle assessment of meat trays made of various packaging materials. *Sustainability* **2019**, *11*, 5324.
48. Horowitz, N.; Frago, J.; Mu, D. Life cycle assessment of bottled water: A case study of Green2O products. *Waste Manag.* **2018**, *76*, 734–743.
49. Islam, M.; Uddin, M.J.; Alshehri, K. Plastic Waste and Carbon Footprint Generation Due to the Consumption of Bottled Waters in Saudi Arabia. *Res. Dev. Mater. Sci.* **2018**, *5*. https://doi.org/10.31031/RDMS.2018.05.000604.
50. Lee, R.P.; Meyer, B.; Huang, Q.; Voss, R. Sustainable waste management for zero waste cities in China: Potential, challenges and opportunities. *Clean Energy* **2020**, *4*, 169–201. https://doi.org/10.1093/ce/zkaa013.
51. Awasthi, A.K.; Cheela, V.R.S.; D'Adamo, I.; Iacovidou, E.; Islam, M.R.; Johnson, M.; Miller, T.R.; Parajuly, K.; Parchomenko, A.; Radhakrishan, L.; et al. Zero waste approach towards a sustainable waste management. *Environ. Dev. Sustain.* **2021**, *3*, 100014. https://doi.org/10.1016/j.resenv.2021.100014.
52. Hao, F. Waste Ban Forces Unlicensed Recyclers to Clean Up Act, 2018, https://chinadialogue.net/en/business/10438-waste-ban-forces-unlicensed-recyclers-to-clean-up-act/ (accessed on 30 May, 2021).
53. Council, C.S. China: National Plan on Banning "Foreign Garbage" and Reducing Solid Waste Imports, 2017, https://www.loc.gov/item/global-legal-monitor/2017-08-08/china-national-plan-on-banning-foreign-garbage-and-reducing-solid-waste-imports/ (accessed on 30 May, 2021).

54. Kuczenski, B.; Geyer, R. *Life Cycle Assessment of Polyethylene Terephthalate (PET) Beverage Bottles Consumed in the State of California*; DRRR-2014-1487; California Department of Resources Recycling and Recovery 14 February, 2011.
55. Coelho, T.M.; Castro, R.; Gobbo, J.A., Jr. PET containers in Brazil: Opportunities and challenges of a logistics model for post-consumer waste recycling. *Resour. Conserv. Recycl.* **2011**, *55*, 291–299.
56. Ma, Z.; Ryberg, M.W.; Wang, P.; Tang, L.; Chen, W.-Q. China's Import of Waste PET Bottles Benefited Global Plastic Circularity and Environmental Performance. *ACS Sustain. Chem.* **2020**, *8*, 16861–16868.
57. Stanway, D. China to force firms to report use of plastic in new recycling push. *World News*, 30 November, 2020.
58. ENF. Plastic Recycling Plants in China, 2021, https://www.enfrecycling.com/directory/plastic-plant/China (accessed on 30 May, 2021).
59. Commission, E. *Enviroment*; Waste Framework Directive, https://ec.europa.eu/environment/topics/waste-and-recycling/waste-framework-directive_en
60. Bartl, A. The EU Circular Economy Package: A genius programme or an old hat? *Waste Manag. Res.* **2018**, *36*, 309–310. https://doi.org/10.1177/0734242X18755022.
61. Hui, Y.; Li'ao, W.; Fenwei, S.; Gang, H. Urban solid waste management in Chongqing: Challenges and opportunities. *Waste Manag.* **2006**, *26*, 1052–1062.
62. Furukawa, M.; Kawakami, N.; Tomizawa, A.; Miyamoto, K. Efficient degradation of poly (ethylene terephthalate) with Thermobifida fusca cutinase exhibiting improved catalytic activity generated using mutagenesis and additive-based approaches. *Sci. Rep.* **2019**, *9*, 16038.
63. Quartinello, F.; Vajnhandl, S.; Volmajer Valh, J.; Farmer, T.J.; Vončina, B.; Lobnik, A.; Herrero Acero, E.; Pellis, A.; Guebitz, G.M. Synergistic chemo-enzymatic hydrolysis of poly (ethylene terephthalate) from textile waste. *Microb Biotechnol* **2017**, *10*, 1376–1383.
64. Charpentier Poncelet, A.; Helbig, C.; Loubet, P.; Beylot, A.; Muller, S.; Villeneuve, J.; Laratte, B.; Thorenz, A.; Tuma, A.; Sonnemann, G. Life cycle impact assessment methods for estimating the impacts of dissipative flows of metals. *J. Ind. Ecol.* **2021**, *25*, 1177–1193. https://doi.org/10.1111/jiec.13136.
65. Kayo, C.; Tojo, S.; Iwaoka, M.; Matsumoto, T. Evaluation of Biomass Production and Utilization Systems. In *Research Approaches to Sustainable Biomass Systems*; 2014; pp. 309–346. https://doi.org/10.1016/B978-0-12-404609-2.00014-3.
66. Jolliet, O.; Margni, M.; Charles, R.; Humbert, S.; Payet, J.; Rebitzer, G.; Rosenbaum, R. IMPACT 2002+: A new life cycle assessment methodology. *Int. J. Life Cycle Assess.* **2003**, *8*, 324–330. https://doi.org/10.1007/BF02978505.
67. Guinee, J. Handbook on Life Cycle Assessment. An Operational Guide to the ISO Standards. *Int. J. Life Cycle Assess.* **2001**, *7*, 311–313. https://doi.org/10.1007/BF02978897.
68. Laurent, A.; Weidema, B.P.; Bare, J.; Liao, X.; de Souza, D.M.; Pizzol, M.; Sala, S.; Schreiber, H.; Thonemann, N.; Verones, F. Methodological review and detailed guidance for the life cycle interpretation phase. *J. Ind. Ecol.* **2020**, *24*, 986–1003. https://doi.org/10.1111/jiec.13012.
69. Matuštík, J.; Kočí, V. What is a footprint? A conceptual analysis of environmental footprint indicators. *J. Clean. Prod.* **2021**, *285*, 124833.
70. de Mello Soares, C.T.; Ek, M.; Östmark, E.; Gällstedt, M.; Karlsson, S. Recycling of multi-material multilayer plastic packaging: Current trends and future scenarios. *Resour. Conserv. Recycl.* **2022**, *176*, 105905.
71. Bach, V.; Minkov, N.; Finkbeiner, M. Assessing the Ability of the Cradle to Cradle Certified™ Products Program to Reliably Determine the Environmental Performance of Products. *Sustainability* **2018**, *10*, 1562. https://doi.org/10.3390/su10051562.
72. Lozano, S.; Iribarren, D.; Moreira, M.; Feijoo, G. A joint application of Life Cycle Assessment and Data Envelopment Analysis. 2003.
73. Gómez, I.D.L.; Escobar, A.S. The dilemma of plastic bags and their substitutes: A review on LCA studies. *Sustain. Prod. Consum.* **2022**, *30*, 107–116. https://doi.org/10.1016/j.spc.2021.11.021.
74. Pajula, T.; Behm, K.; Vatanen, S.; Saarivuori, E. Managing the Life Cycle to Reduce Environmental Impacts. In *Dynamics of Long-Life Assets: From Technology Adaptation to Upgrading the Business Model*; Grösser, S.N., Reyes-Lecuona, A., Granholm, G., Eds.; Springer International Publishing: Cham, Switherland, 2017; pp. 93–113. https://doi.org/10.1007/978-3-319-45438-2_6.
75. Perera, F. Pollution from Fossil-Fuel Combustion is the Leading Environmental Threat to Global Pediatric Health and Equity: Solutions Exist. *Int. J. Environ. Res. Public Health* **2017**, *15*, 16. https://doi.org/10.3390/ijerph15010016.
76. Ali, H.; Khan, E.; Ilahi, I. Environmental Chemistry and Ecotoxicology of Hazardous Heavy Metals: Environmental Persistence, Toxicity, and Bioaccumulation. *J. Chem.* **2019**, *2019*, 6730305. https://doi.org/10.1155/2019/6730305.

77. Xue, T.; Zhu, T.; Zheng, Y.; Zhang, Q. Declines in mental health associated with air pollution and temperature variability in China. *Nat. Commun.* **2019**, *10*, 2165.
78. Thrasher, J.D.; Kilburn, K.H. Embryo Toxicity and Teratogenicity of Formaldehyde. *Arch. Environ. Health Int. J.* **2001**, *56*, 300–311. https://doi.org/10.1080/00039890109604460.
79. Stevens, C.J.; David, T.I.; Storkey, J. Atmospheric nitrogen deposition in terrestrial ecosystems: Its impact on plant communities and consequences across trophic levels. *Funct. Ecol.* **2018**, *32*, 1757–1769. https://doi.org/10.1111/1365-2435.13063.
80. Andersen, S.O.; Halberstadt, M.L.; Borgford-Parnell, N. Stratospheric ozone, global warming, and the principle of unintended consequences—An ongoing science and policy success story. *J. Air Waste Manag. Assoc.* **2013**, *63*, 607–647. https://doi.org/10.1080/10962247.2013.791349.
81. Malone, T.C.; Newton, A. The Globalization of Cultural Eutrophication in the Coastal Ocean: Causes and Consequences. *Front. Mar. Sci.* **2020**, *7*, 670.
82. Wang, S.; Zhu, Y.; Wu, Q. Design of a sorting and recycling system for post-consumer bottles. In Proceedings of the 2022 International Seminar on Computer Science and Engineering Technology (SCSET), Indianapolis, IN, USA, 8–9 January 2022; pp. 281–286.
83. Linzner, R.; Salhofer, S. Municipal solid waste recycling and the significance of informal sector in urban China. *Waste Manag. Res.* **2014**, *32*, 896–907.
84. Steuer, B. Is China's regulatory system on urban household waste collection effective? An evidence-based analysis on the evolution of formal rules and contravening informal practices. *J. Chin. Gov.* **2017**, *2*, 411–436. https://doi.org/10.1080/23812346.2017.1379166.
85. Zhang, H.; Wen, Z.-G. The consumption and recycling collection system of PET bottles: A case study of Beijing, China. *Waste Manag.* **2014**, *34*, 987–998.
86. Chi, X.; Streicher-Porte, M.; Wang, M.Y.L.; Reuter, M.A. Informal electronic waste recycling: A sector review with special focus on China. *Waste Manag.* **2011**, *31*, 731–742.
87. Europe, P.; EPRO. Plastics—The Facts 2020, https://plasticseurope.org/knowledge-hub/plastics-the-facts-2020/ (accessed on 25 October, 2021).
88. Smithers. Global PET Packaging Demand to Reach $44.1 Billion in 2020 Says Smithers Report, 2020, https://www.smithers.com/resources/2020/sept/global-pet-packaging-demand-to-reach-$44-1-billion (accessed on 28 October, 2021).
89. Rosenboom, J.-G.; Langer, R.; Traverso, G. Bioplastics for a circular economy. *Nat. Rev. Mater.* **2022**, *7*, 117–137. https://doi.org/10.1038/s41578-021-00407-8.
90. Suwanmanee, U.; Leejarkpai, T.; Mungcharoen, T., Assessment the environmental impacts of polylactic acid/starch and polyethylene terephthalate boxes using life cycle assessment methodology: Cradle to waste treatments. *J Biobased Mater* **2013**, *7*, 259-266.
91. Nie, Y., Development and prospects of municipal solid waste (MSW) incineration in China. *Front. Env. Sci Eng. China* **2008**, *2*, 1-7.
92. Xu, W. L.; Liu, J. H., Status and development prospect on municipal solid waste incineration technology in our country. *China Environ. Prot. Ind.* **2007**, *11*, 24-29.
